# Cryptic Species Due to Hybridization: A Combined Approach to Describe a New Species (*Carex*: Cyperaceae)

**DOI:** 10.1371/journal.pone.0166949

**Published:** 2016-12-14

**Authors:** Enrique Maguilla, Marcial Escudero

**Affiliations:** 1 Departamento de Biología Molecular e Ingeniería Bioquímica, Universidad Pablo de Olavide, Seville, Spain; 2 Departamento de Biología Vegetal y Ecología, Universidad de Sevilla, Seville, Spain; National Cheng Kung University, TAIWAN

## Abstract

Disappearance of diagnostic morphological characters due to hybridization is considered to be one of the causes of the complex taxonomy of the species-rich (ca. 2000 described species) genus *Carex* (Cyperaceae). *Carex furva s*.*l*. belongs to section *Glareosae*. It is an endemic species from the high mountains of the Iberian Peninsula (Spain and Portugal). Previous studies suggested the existence of two different, cryptic taxa within *C*. *furva s*.*l*. Intermediate morphologies found in the southern Iberian Peninsula precluded the description of a new taxa. We aimed to determine whether *C*. *furva s*.*l*. should be split into two different species based on the combination of morphological and molecular data. We sampled ten populations across its full range and performed a morphological study based on measurements on herbarium specimens and silica-dried inflorescences. Both morphological and phylogenetic data support the existence of two different species within *C*. *furva s*.*l*. Nevertheless, intermediate morphologies and sterile specimens were found in one of the southern populations (Sierra Nevada) of *C*. *furva s*.*l*., suggesting the presence of hybrid populations in areas where both supposed species coexist. Hybridization between these two putative species has blurred morphological and genetic limits among them in this hybrid zone. We have proved the utility of combining molecular and morphological data to discover a new cryptic species in a scenario of hybridization. We now recognize a new species, *C*. *lucennoiberica*, endemic to the Iberian Peninsula (Sierra Nevada, Central system and Cantabrian Mountains). On the other hand, *C*. *furva s*.*s*. is distributed only in Sierra Nevada, where it may be threatened by hybridization with *C*. *lucennoiberica*. The restricted distribution of both species and their specific habitat requirements are the main limiting factors for their conservation.

## Introduction

Around 2000 species have been recognized in the genus *Carex* L. (Cyperaceae; [1–3]) which is one of the largest genera among the angiosperms as the result of a relatively fast radiation mainly in temperate areas of the Northern Hemisphere [4,5]. The study of the genus as a whole has derived in taxonomic rearrangements based on phylogenetic studies [2]. Incomplete phylogenies of the genus *Carex* (e.g. [6,7]), or focusing on infrageneric taxa (subgenera or sections) (e.g. [8–10]), the restricted geographic coverage of the studies, mostly focused on Europe and North America, and the historical non-natural classifications of the genus, are some of the causes that have hampered an extensive revision of the genus [11]. Moreover, hybridization in *Carex* has been proposed to limit taxonomic delimitation of species [12–14].

Using molecular tools and combinations of different approaches have been demonstrated to be crucial to detect hybrid zones [15] and discover new cryptic species [16–18]. Hybridization and/or introgression cause reticulate evolution [18] which makes the establishment of limits among species difficult.

*Carex* section *Glareosae* G. Don has a circumboreal distribution and constitutes a monophyletic clade comprising 25 currently recognized species [7,14,15]. Species in *Carex* section *Glareosae* have experienced multiple taxonomic rearrangements (e.g. [8,19–21]) due to its remarkable morphological, biogeographic and ecological variability [19–21]. The taxonomic identity of some species within the section is still unclear as it occurs with *C*. *kreczetoviczii* T.V.Egor. [8,19] and *C*. *furva* Webb [22]. The taxonomy of *C*. *furva s*.*l*., endemic to the Iberian Peninsula (Spain and Portugal), has been discussed in the past. This species was considered either a synonym of *C*. *lachenalii* Schkuhr, a subspecies or a variety of this species (see discussion in [22]). Nowadays, it has been broadly demonstrated from a morphological, phylogenetic and cytogenetic point of view, that C. *furva s*.*l*. and *C*. *lachenalii* are different species [8,22].

Within *C*. *furva s*.*l*., different morphogroups were detected by Gay [23] and later by Luceño [22]: one from the southern Iberian Peninsula, and another group constituted by central and northern populations of the species in the Iberian Peninsula. While Gay [23] considered central and northern morphotype of *C*. *furva s*.*l*. to be a subspecies of *C*. *lachenalii* [23], Luceño [22] did not. Intermediate morphologies in the southern Iberian Peninsula as well as the continuum of variation of diagnostic characters prevented him the consideration of this taxon. Nowadays, both morphotypes are considered a single species, *C*. *furva* [3,22]. Maguilla et al. [8] suggested the existence of an incipient speciation event involving these populations. Morphological data supports the presence of intermediate morphologies in the southern Iberian Peninsula [22] which could reflect hybridization processes between different taxa. Hybridization can act as a homogenization force of both genetic and morphological traits among species [24,25], and consequently *C*. *furva s*.*l*. could be hiding a cryptic taxon. Hybrids in *Carex* section *Glareosae* have been described to be mostly sterile. In fact, hybrid speciation seems not to be a major evolutionary force for *Carex* in general [12–14] or specifically for this section [8,19,20,26,27]. Previous studies by Maguilla et al. [8] and Luceño [22] suggest that hybridization could be avoiding the detection of a cryptic species within *C*. *furva s*.*l*.

The aim of this study is to delimitate the morphological variability of both previously detected genetic entities within *C*. *furva s*.*l*. and to decide whether to consider a new species, subspecies or variety. We have performed a combined approach based on statistical analyses of morphological and phylogenetic data to discriminate taxa that could have remained cryptic due to the existence of morphologically intermediate individuals.

## Materials and Methods

### Study species

**Fig 1:**
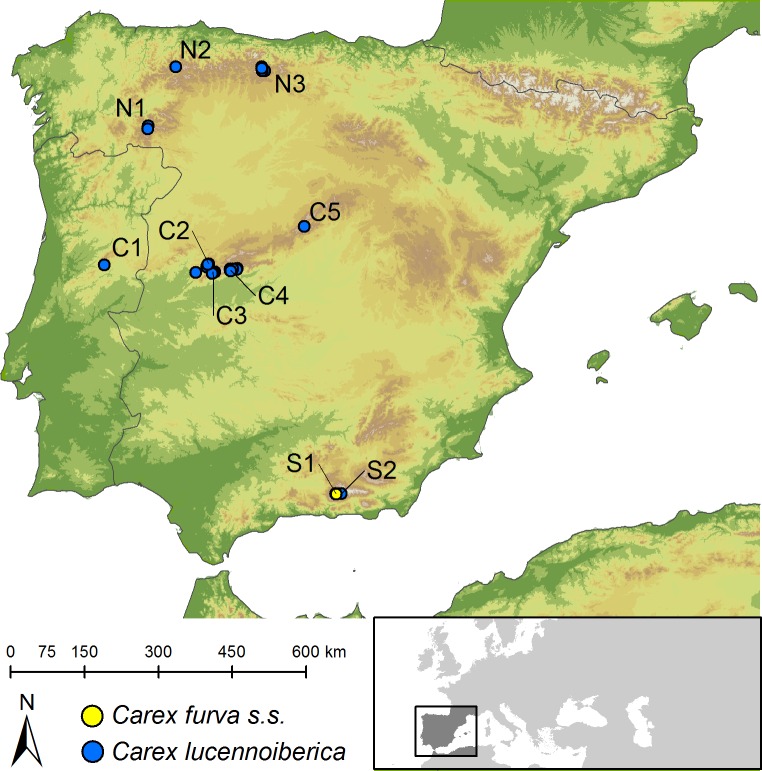
Distribution map of *C*. *furva s*.*s*. and *C*. *lucennoiberica* based on examined specimens on herbaria collections and field trip records. Codes indicate sampled populations as follows: C1 = Serra da Estrela; C2 = Sierra de Béjar; C3 = Sierra del Barco; C4 = Picos de Gredos; C5 = Sierra de Guadarrama; N1 = Sierra Segundera; N2 = Sierra del Cornón; N3 = Fuentes Carrionas (Curavacas); S1 (*C*. *furva s*.*s*.) and S2 (hybrid) = Sierra Nevada. Created using country borders from Brummitt et al. [62] and elevation data from CGIAR [63] under a CC BY license, with permission from CGIAR, original copyright 2008.

*Carex furva s*.*l*. is a species endemic to the Iberian Peninsula that belongs to *Carex* section *Glareosae*. Previous phylogenetic studies have shown this species to be monophyletic [8]. The highly specific ecological requirements of soils on acid bedrocks and very cool environments [28] explain the distribution of the species in the highest mountains of the Iberian Peninsula, never occurring below 1800 m.a.s.l. [28]. Currently, *C*. *furva s*.*l*. has been found only in seven mountain ranges in the Iberian Peninsula (Sierra Segundera, Sierra del Cornón, Fuentes Carrionas, Sierra de Gredos, Sierra de Guadarrama and Sierra Nevada in Spain, plus Serra da Estrela in Portugal; [28,29]). Our sampling exhaustively covered the full range of the species (Fig 1). Field collecting permits were provided by Instituto de Conservação da Naturaleza e da Biodiversidade (ICNB, Portugal), Junta de Andalucía (Department of Environment, Spain) and Community of Madrid (Department of Environment, Local Government and Territorial Planning, Spain). Destructive sampling for DNA extraction was provided by UPOS herbarium.

### Morphometric study

**Fig 2:**
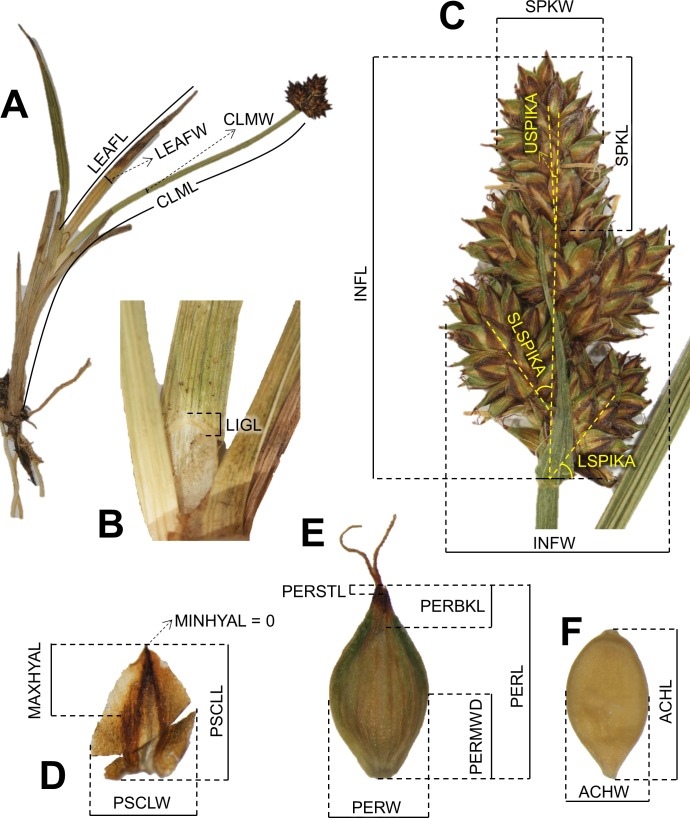
Representation of measured morphological variables in a specimen. (A) General aspect; (B) ligule; (C) inflorescence; (D) female glume; (E) utricle; (F) achene. Photographs A, B and D correspond to *C*. *furva s*.*s*. and C, E and F to *C*. *lucennoiberica*. Meaning of the variables as described in Table 1.

Sampling for morphological study included 43 herbarium specimens (M, SEV and UPOS herbaria [30]) and samples of inflorescences of 60 specimens collected in field trips and preserved in silica-gel. Thus, a total of 103 individual specimens were measured including six to 21 individuals per population (ten sampled populations) to reflect the full range of morphological variation in the species. The exceptions were populations in Fuentes Carrionas (N3; Fig 1) where only one specimen was available at UPOS herbarium, and population from Serra da Estrela (C1; Fig 1), with only one surviving individual [29]. Type specimen of *C*. *furva s*.*s*. [31] was visually inspected but not included in any of the analyses. Twenty-seven morphological variables were selected and measured based on characters used for the description of species in *Carex* sect. *Glareosae* in different flora ([19–22]; Table 1 and Fig 2). Only one measurement per variable and specimen was taken (avoiding redundancy) from each specimen. We randomly selected a mature shoot per specimen. However, minimum and maximum values for each variable and individual were obtained measuring all mature shoots of each specimen. An ocular micrometer was used for characters shorter than 10 mm, and a 30-cm ruler when larger than 10 mm. Angles were measured with a standard angular encoder. Moreover, three new variables were calculated to represent the shape of the inflorescence and utricles: the ratio inflorescence length: inflorescence width (INFL/INFW), the ratio utricle length: utricle width (PERL/PERW) and the ratio utricle length: distance from the utricle base to its maximum width (PERL/PERMWD; Table 1). Minimum and maximum ranges of culm, leaf, inflorescence and spike lengths and widths were also measured. For the statistical analyses, culm length (CLML), ligule length (LIGL) and leaf characters (ILEAFL, ILEAFW, SLEAFL and SLEAFW; Table 1 and Fig 2) were excluded due to the lack of data in most of the samples from silica-preserved specimens. To avoid redundancy in statistical analyses, ratios (INFL/INFW, PERL/PERW and PERL/PERMWD; Table 1) were used only for species descriptions and not for statistical analyses. Accordingly, a total of 21 variables were included in statistical analyses.

**Table 1:** Morphological variables and descriptions

Variable	Description (units)
CLML	Distance from the base of the culm to the start of the inflorescence (mm)
CLML-max	Maximum culm length in a specimen when more than one fertile and mature culm is present (mm)
CLML-min	Minimum culm length in a specimen when more than one fertile and mature culm is present (mm)
CLMW	Width of the culm in the medial region (mm)
CLMW-max	Maximum culm width in a specimen when more than one fertile and mature culm is present (mm)
CLMW-min	Minimum culm width in a specimen when more than one fertile and mature culm is present (mm)
ILEAFL	Distance from the base to the tip of the inferior leaf (mm)
ILEAFW	Width of the inferior leaf in the medial portion (mm)
SLEAFL	Distance from the base to the tip of the superior leaf (mm)
SLEAFW	Width of the superior leaf in the medial portion (mm)
LEAFL-max	Maximum leaf length in a specimen (mm)
LEAFL-min	Minimum leaf length in a specimen (mm)
LEAFW-max	Maximum leaf width in a specimen (mm)
LEAFW-min	Minimum leaf width in a specimen (mm)
LIGL	Maximum ligule length (mm)
INFL	Distance from the base of the inflorescence to the bottom of the uppermost utricle beak (mm)
INFL-max	Maximum inflorescence length in a specimen when more than one fertile and mature culm is present (mm)
INFL-min	Minimum inflorescence length in a specimen when more than one fertile and mature culm is present (mm)
INFW	Maximum width of the inflorescence in horizontal, from the bases of the utricle beaks (mm)
INFW-max	Maximum inflorescence width in a specimen when more than one fertile and mature culm is present (mm)
INFW-min	Minimum inflorescence width in a specimen when more than one fertile and mature culm is present (mm)
INFL/INFW	Ratio inflorescence length: inflorescence width (mm)
SPKN	Number of spikes in the inflorescence (entire number)
SPIKL	Distance from the base of the apical spike to the bottom of the uppermost utricle beak (mm)
SPIKL-max	Maximum spike length in a specimen (mm)
SPIKL-min	Minimum spike length in a specimen (mm)
SPIKW	Maximum width of the apical spike excluding utricle beaks (mm)
SPIKW-max	Maximum spike width in a specimen (mm)
SPIKW-min	Minimum spike width in a specimen (mm)
LSPIKA	Angle of the lowermost spike of the inflorescence relative to the culm (degrees)
SLSPIKA	Angle of the second lower spike–from the bottom–of the inflorescence relative to the culm (degrees)
USPIKA	Angle of the uppermost spike of the inflorescence relative to the culm (degrees)
PSCLL	Maximum glume length of the medial point of the spike (mm)
PSCLW	Maximum glume width of the medial point of the spike (mm)
MAXHYAL	Length of the widest hyaline margin in female glumes (mm)
MINHYAL	Length of the narrowest hyaline margin in female glumes (mm)
PERL	Maximum length of the utricle from the base, including the beak (mm)
PERW	Maximum width of the utricle (mm)
PERL/PERW	Ratio utricle length: utricle width
PERMWD	Distance from the maximum width to the base of the utricle (mm)
PERL/PERMWD	Ratio utricle length: distance from the base to the maximum width distance of the utricle
PERBKL	Distance from distal point of the utricle to the distal point of the achene (mm)
PERSTL	Distance from the distal point of the utricle beak to the end of the abaxial suture (mm)
PERIGTHN	Number of teeth in the utricle beak (entire number)
ACHL	Maximum achene length (mm)
ACHW	Maximum achene width (mm)

Principal component analyses (PCA) were performed in IBM SPSS Statistics v.20 (IBM Inc., Chicago, IL, USA) rescaling variables to unit variance. We followed an analytical procedure based on Jiménez-Mejías et al. [32]. Characters reaching more than 0.6 of weigh in principal components as well as eigenvalues greater than 1 were used to perform a second PCA. Kaiser-Meyer-Olkin’s measure of sampling adequacy (KMO) and Bartlett’s test of sphericity were also estimated to evaluate the suitability of the data for finding structure in both approaches. PCAs were performed twice, including and excluding putative hybrids. Discriminant function analysis (DFA) was then performed in IBM SPSS Statistic v.20, using all variables included in the first PCA approach, to evaluate for taxonomic significance of two morphogroups as described in Valcárcel & Vargas [33], considering as potentially significant those groups correctly classified in 80% of excluded cases as established in Jiménez-Mejías et al. [32]. We randomly selected 70% of all samples to perform the DFA using a cross-validation of the model over these samples. Then, the remaining 30% was used for an additional validation. Based on the finding of intermediate individuals in Sierra Nevada by Luceño [22], populations S1 and S2 from Sierra Nevada (Fig 1) were studied very carefully. According to our own results (see below) we removed the population S2 from Sierra Nevada (Fig 1) from the subsequent analyses. Thus, we classified population S2 entirely as hybrid based on the consideration by Luceño [22] as intermediate morphology and the presence of sterile individuals detected (pers. obs.). All individuals from the hybrid population (S2; Fig 1) were unselected for the DFA and used only for the validation of the model using unselected cases, to test the placement of each individual from this population in any of the two groups. Additionally, univariate analyses were performed based on groups detected in the PCA to evaluate the characters that best allow the discrimination between the two species/taxa/morphologies. The Shapiro Wilk normality test showed non-normal distribution for most of the variables. The violation of the normality criteria in PCA and DFA analyses can be assumed when considering results as indicative and not a final evidence for taxonomic decisions [33], as these analyses are almost insensitive to such violation [34]. Then, variation between groups was evaluated through a Kruskal-Wallis one-way ANOVA and a post-hoc Mann-Whitney U pairwise test to assess for significant differences between morphogroups for each character.

### Phylogenetic analyses

Eight of our already published [8] sequences of the ITS, ETS and G3PDH nrDNA regions of *C*. *furva* s.l. as well as the *mat*K cpDNA region were used for this study including two samples from southern Iberian Peninsula and six samples from central-northern populations (S1 File). Given that we aim to test for phylogenetic significance for the two detected morphogroups (see results), the population considered hybrid (S2; Fig 1) was excluded because we could not assign it to any of the two morphogroups. Additionally, sequences of these four markers were downloaded for species in *Carex* section *Glareosae*. According to the phylogeny in Maguilla et al. [8], the following outgroup species were included: *Carex arctiformis* Mack., *C*. *billingsii* (O.W.Knight) C.D.Kirschb., *C*. *bonanzensis* Britton, *C*. *brunnescens*, *C*. *canescens* L., *C*. *diastena* V.I.Krecz., *C*. *glareosa* Schkuhr ex Wahlenb., *C*. *heleonastes* Ehrh. ex L.f., *C*. *kreczetoviczii* T.V.Egor., *C*. *lachenalii*, *C*. *lapponica* O.Lang, *C*. *loliacea* L., *C*. *mackenziei* V.I.Krecz., *C*. *marina* Dewey, *C*. *nemurensis* Franch., *C*. *praeceptorum* Mack., *C*. *pseudololiacea* F.Schmidt, *C*. *tenuiflora* Wahlenb., *C*. *traiziscana* F.Schmidt. *C*. *trisperma* Dewey and *C*. *ursina* Dewey (S1 File). Sequences were automatically aligned using MUSCLE [35] and concatenated to be analyzed using Bayesian inference (BI) and maximum likelihood (ML) as performed in Maguilla et al. [8]. Substitution models were calculated for each DNA region in jModelTest v.2.1.3 [36] and selected based on the Akaike’s Information Criterion weights (AICw [37]). Gaps were encoded based on the “simple indel coding” criterion described by Simmons and Ochoterena [38] and analyzed using a F81-like substitution model as suggested by MrBayes manual [39].

### Nomenclature

The electronic version of this article in Portable Document Format (PDF) in a work with an ISSN or ISBN will represent a published work according to the International Code of Nomenclature for algae, fungi, and plants, and hence the new names contained in the electronic publication of a PLOS article are effectively published under that Code from the electronic edition alone, so there is no longer any need to provide printed copies.

In addition, new names contained in this work have been submitted to IPNI, from where they will be made available to the Global Names Index. The IPNI LSIDs can be resolved and the associated information viewed through any standard web browser by appending the LSID contained in this publication to the prefix http://ipni.org/. The online version of this work is archived and available from the following digital repositories: PubMed Central, LOCKSS.

## Results

### Morphometric study

**Fig 3:**
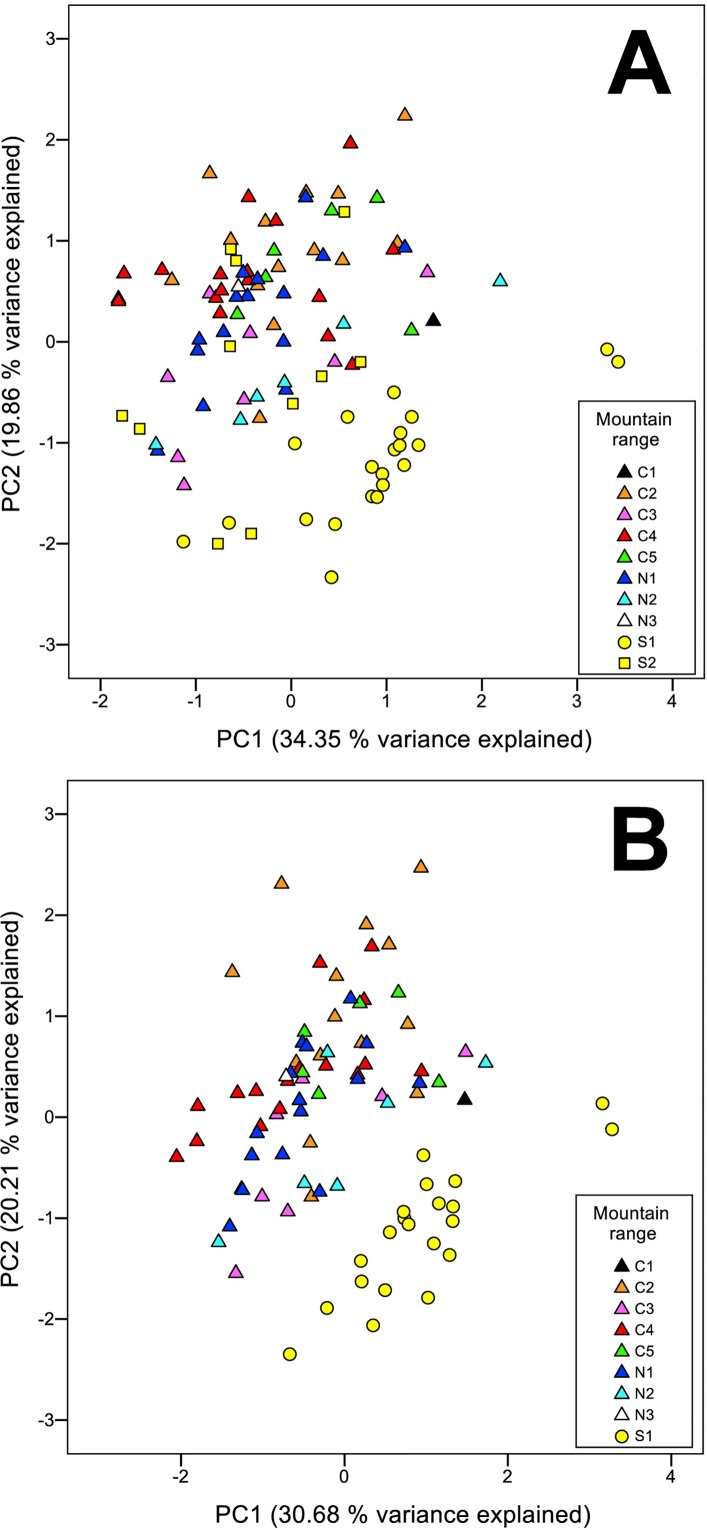
**Principal Component Analysis (PCA) scatter plot of the first two principal components: A. including hybrid population; B. excluding hybrids.** Only selected variables were used (nine when including hybrid populations, and 11 when excluded). Circles represent *Carex furva s*.*s*., triangles for *C*. *lucennoiberica*, and squares for specimens of the hybrid population. Colors indicate the mountain range where the specimens were collected, where C1 = Serra da Estrela; C2 = Sierra de Béjar; C3 = Sierra del Barco; C4 = Picos de Gredos; C5 = Sierra de Guadarrama; N1 = Sierra Segundera; N2 = Sierra del Cornón; N3 = Fuentes Carrionas (Curavacas); S1 (*C*. *furva s*.*s*.) and S2 (hybrid) = Sierra Nevada.

The scatter plot of the two main principal components from the analysis using all variables (S2 File), as well as the analysis performed using only nine and 11 variables (including and excluding the intermediate population respectively; Fig 3 and S2 File), show two clearly differentiated morphogroups (PC1; 34.35% variance explained using selected variables when including the intermediate population; 30.68% when excluding the intermediate) and two (PC2; 19.86% variance explained using selected variables including intermediates; 20.21% when excluded). The first group was formed by central and northern populations of *C*. *furva s*.*l*. whereas the second was constituted by population S1 from the southern Iberian Peninsula (Figs 1 and 3), which definitely fits with the morphology of the type specimen of *C*. *furva s*.*s*. [31]. Individuals belonging to the population considered as hybrid were dispersed in the scatterplot of the principal components one and two (PC1 and PC2), with individuals nested in both morphogroups (Fig 3A and S2 File). Once delimited both morphogroups, DFA analysis correctly classified 100% of the original selected cases and 92.2% in the cross validation (S3 File). The analysis of unselected cases retrieved a 92% of cases correctly classified. Hybrid individuals are considered to belong to the central-northern morphogroup in 72.7% of cases, whereas the remaining 27.3% are considered morphologically similar to the southern group (S3 File).

When compared with the type specimen of *C*. *furva* Webb [31], every sampled individual in population S1 fits definitively with this type material, whereas individuals from population S2 (Fig 1) look intermediate between Webb’s *C*. *furva* and northern morphology of *C*. *furva s*.*l*.

Despite some overlap in the range of many characters in both morphogroups, the ANOVA and Mann-Whitney U test retrieved significant differences (P-value <0.01) in ten out of 21 characters: ACHL, INFL, INFW, LSPIKA, PERL, PERBKL, PERSTL, SLSPIKA, SPIKW and SPKN (Table 1, Fig 2 and S4 File).

### Phylogenetic analyses

Concatenated and aligned matrix of the ETS, ITS, G3PDH and *mat*K DNA regions consisted of 29 sequences (S1 File) and 2212 sites which include the codification of four indels. The nucleotide substitution model that best fits each DNA region based on jModelTest results were: GTR+I (AICw = 0.4309) for ETS, GTR+G (AICw = 0.7193) for ITS, HKY (AICw = 0.4217) for G3PDH and GTR+I (AICw = 0.3015) in the case of the *mat*K cpDNA region.

**Fig 4:**
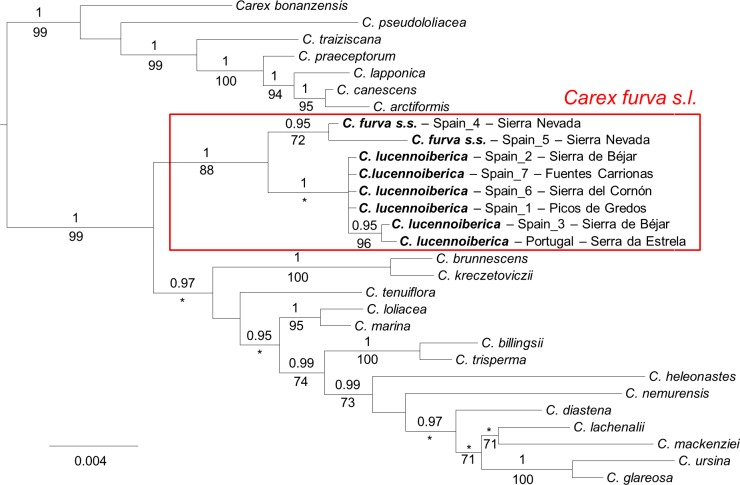
Majority-rule consensus tree from Bayesian inference analysis of the concatenated matrix of nrDNA regions ETS, ITS, G3PDH and cpDNA region *mat*K. Posterior probabilities (PP, only if higher than 0.9) from the Bayesian analysis and bootstrap values (if > 70%) from the maximum likelihood analysis are shown above and below branches, respectively. Lack of support in only one analysis is represented by asterisks. Tip labels indicate species name. In the case of *C*. *furva s*.*s*. and *C*. *lucennoiberica*, we have also included sampling locality. Red square represents the boundaries of *C*. *furva s*.*l*. Scale bar indicates substitutions per site.

Bayesian inference and ML analyses supported the monophyly of *C*. *furva s*.*l*. with 1.0 posterior probability (PP) and 88% bootstrap support (BS) respectively (Fig 4). Within *C*. *furva s*.*l*. two main clades were significantly supported: one grouping southern individuals (0.95 PP / 72% BS) and the other formed by central-northern individuals (1.0 PP). The central-northern clade comprised also a subclade (0.95 PP / 96% BS; Fig 4) represented by one individual from Spain (Sierra de Béjar, population C2; Fig 1) and one individual from Portugal (Serra da Estrela, population C1; Fig 1).

## Discussion

### A new species hidden within *C*. *furva s*.*l*.

**Fig 5:**
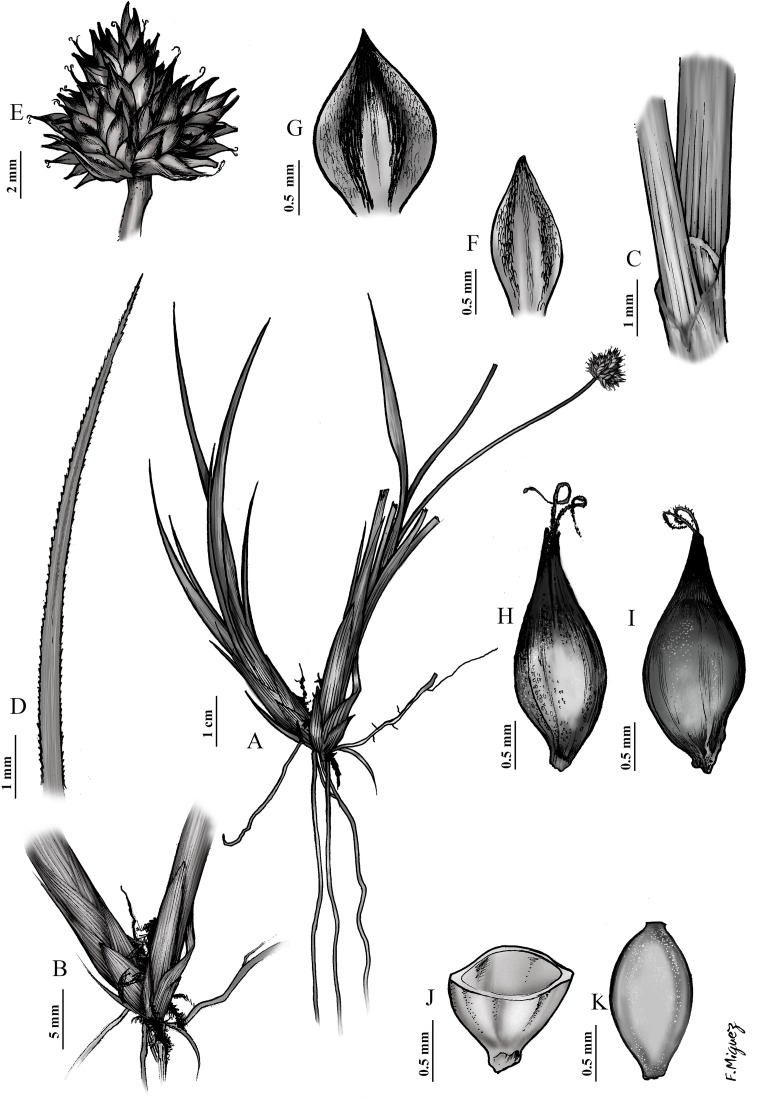
Botanical illustration of *Carex furva* Webb. SPAIN: Granada, Sierra Nevada, Capileira, Sierra Nevada National Park. 08 August 2013. E. Maguilla (31EMS13(15)) & J. M. G. Cobos. UPOS-5132. (A) General aspect; (B) culm base; (C) ligule; (D) leaf appex; (E) inflorescence; (F) male glume; (G) female glume; (H) utricle, abaxial view; (I) utricle, adaxial face; (J) utricle, cross-section; (K) achene.

**Fig 6:**
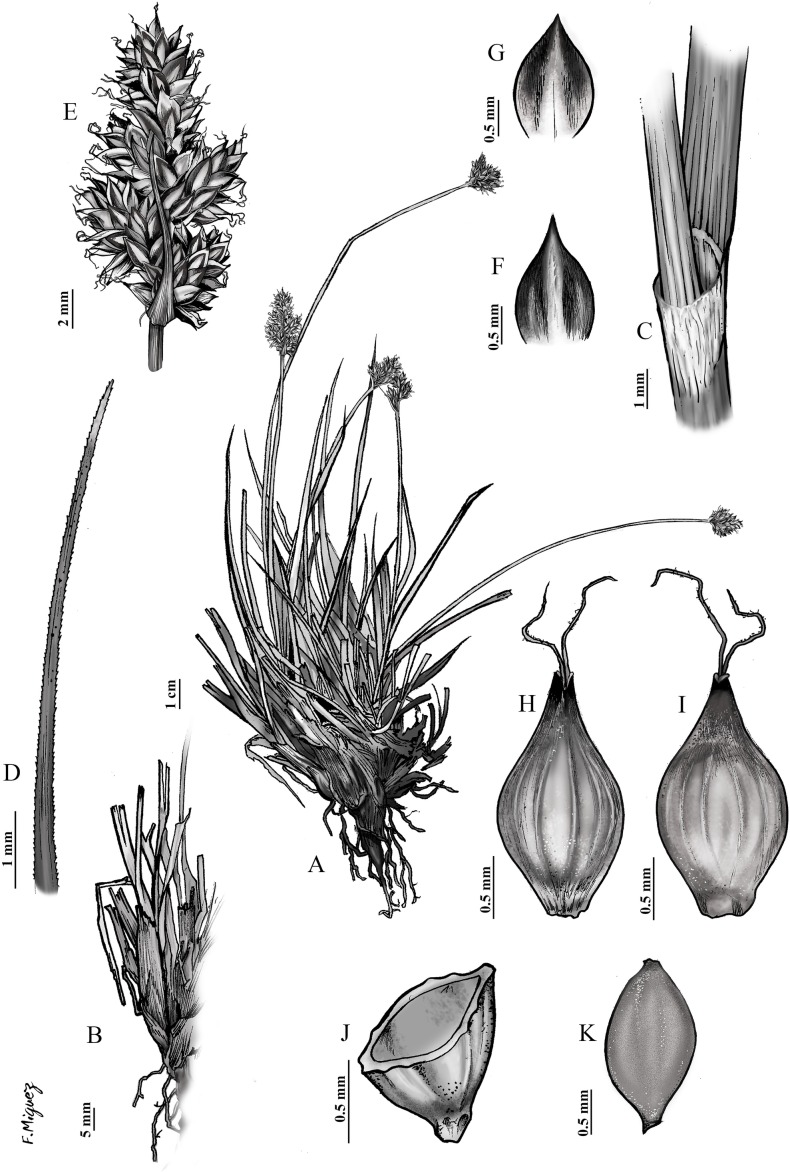
Botanical illustration of *Carex lucennoiberica* Maguilla & M. Escudero. **Paratype.** SPAIN: Madrid, Sierra de Guadarrama, Rascafría, Sierra de Guadarrama Nacional Park. 22 August 2013. E. Maguilla (35EMS13(5)) & T. Villaverde. UPOS-5141. (A) General aspect; (B) culm base; (C) ligule; (D) leaf appex; (E) inflorescence; (F) male glume; (G) female glume; (H) utricle, abaxial view; (I) utricle, adaxial face; (J) utricle, cross-section; (K) achene.

**Fig 7:**
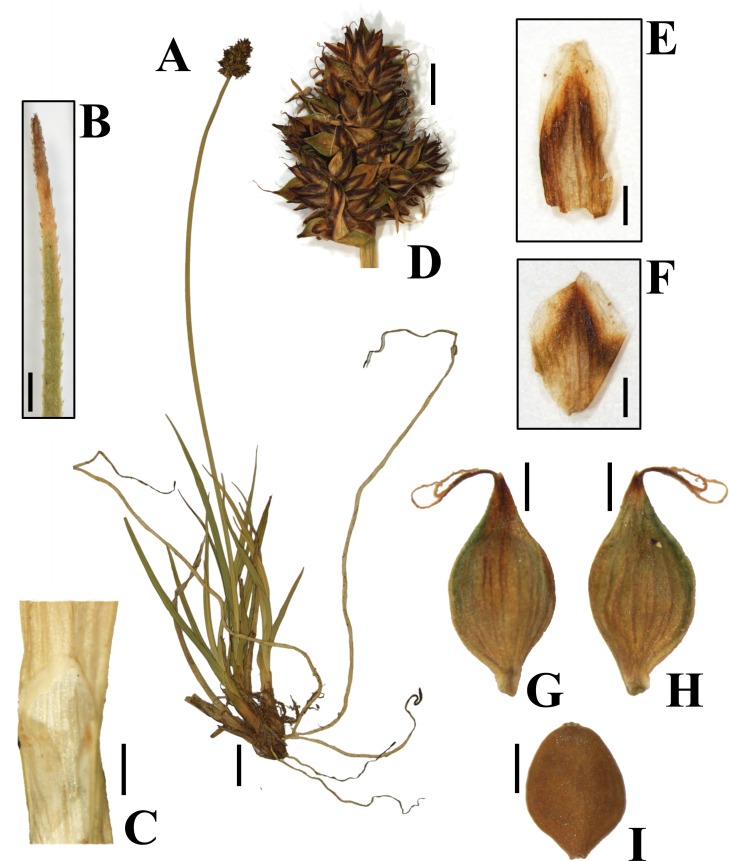
*Carex lucennoiberica* Maguilla & M. Escudero. **Holotype.** SPAIN: Ávila, Sierra de Béjar, arroyo Malillo. 07-August-2010. M. Luceño (21ML10), P. Jiménez-Mejías & M. González. UPOS-4319. (A) General aspect; (B) leaf apex; (C) ligule; (D) inflorescence; (E) male glume; (F) female glume; (G) utricle, abaxial view; (H) utricle, adaxial view; (I) achene. Scale bars: A = 1 mm; B = 0.5 mm; C = 1 mm; D = 2 mm; E-I = 0.5 mm.

Consideration of two different species within *C*. *furva s*.*l*. was precluded based only on more traditional morphological studies because of the existence of morphologically intermediate individuals in the southern Iberian Peninsula [22]. In a molecular approach excluding the inferred hybrid population (Fig 4), two out of the three monophyletic clades significantly supported within *C*. *furva s*.*l*. have both geographical (Fig 1) and morphological (Fig 3) significance. Moreover, DFA analyses correctly classified 92% of unselected cases (S3 File), and ten out of the 21 measured characters presented significant differences between groups based on Mann-Whitney U test (S4 File). These evidences are enough for the consideration of two different species: *C*. *furva s*.*s*. (Fig 5) and a new species, *C*. *lucennoiberica* (Figs 6 and 7). This clear morphological and genetic differentiation between *C*. *furva s*.*s*. and *C*. *lucennoiberica* (Figs 3 and 4; S2–S4 Files) when excluding hybrid individuals from the analyses is in congruence with the observations by Luceño [22]. The new species fits the criteria of taxonomic [40] and phylogenetic [41,42] species. Moreover, the finding of sterile specimens occurring in the hybrid population (S2; Fig 1) suggests incipient reproductive isolation between *C*. *furva s*.*s*. and *C*. *lucennoiberica*. Therefore, the two species might also fulfill the criteria to be biological species [43,44]. A third significantly supported clade is found within *C*. *furva s*.*l*. (0.95 PP / 96% BS; Fig 4) gathering a sample from Serra da Estrela (C1; Fig 1) and another from Sierra de Béjar (C2; Fig 1). The absence of morphological and/or geographic significance of this group (Fig 3) leads us to suggest the existence of a simple genetic structure within *C*. *lucennoiberica*.

Individuals in the intermediate population share morphological similarities with both species (Fig 3A), although most of them seems to be closer to *C*. *lucennoiberica* (most individuals fall within *C*. *lucennoiberica* morphospace in the PCA (Fig 3A) and 72.7% of individuals were assigned to *C*. *lucennoiberica* morphogroup in the DFA (S3 File)). In addition, the finding of sterile specimens and the classification of less than 80% of cases in one or another group (S3 File), justify the consideration of population S2 (Fig 1) in the southern Iberian Peninsula as hybrid population with morphological affinities to both species. When there is a hybrid zone—as it occurs in *C*. *furva s*.*s*. and *C*. *lucennoiberica*—, studies only based on morphology might fail in finding clear limits among the species involved. Luceño [22], in a morphology-based study without statistical methods behind it, highlighted that *C*. *furva s*.*l*. could constitute two independent biological entities. However, individuals with intermediate morphology found in the south of the Iberian Peninsula (Sierra Nevada) prevented him describing a new species. Descriptions of new *Carex* species based exclusively on morphology have been published recently (e.g. [45,46]). New animal species has even been described based only on molecular data, with neither morphological nor ecological traits differentiating each taxon (e.g. [47]; although similar cases have not been found in plants). Describing new species on the unique base of molecular data has been considered as something to avoid since molecular data should be used as an additional evidence for species delimitation [48,49]. Combined approaches of both morphological and molecular data and statistical analyses of those data are currently the most frequent practice for species delimitation and new species descriptions in botany as well as in zoology (e.g. [50–56]). The combination of morphological and molecular data has been previously shown to be a powerful tool to resolve the taxonomy in *Carex* (e.g. [10,57]). This highlights the utility of combined approaches in the detection and description of cryptic species even in countries or regions where the flora is very well studied and known.

### Occurrence of *C*. *lucennoiberica* in the southern Iberian Peninsula

Whereas *C*. *furva s*.*s* is restricted to Sierra Nevada in the southern Iberian Peninsula (Fig 1), additional studied specimens (see paratypes) revealed that *C*. *lucennoiberica* is restricted to mountains in center-northern Iberian Peninsula (Sierra Segundera, Sierra del Cornón, Fuentes Carrionas, Serra da Estrela, Sierra de Gredos and Sierra de Guadarrama), but also present in the southern Iberian Peninsula (Sierra Nevada, Fig 1). One herbarium specimen in the southern Iberian Peninsula fits morphologically with *C*. *lucennoiberica* (see paratypes), whereas several studied materials from different herbaria present intermediate morphology. The observed intermediate morphologies (Fig 3A) and the existence of sterile specimens (pers. obs.) point to the coexistence of *C*. *lucennoiberica* and *C*. *furva s*.*s*. in the southern Iberian Peninsula, suffering active hybridization. Moreover, the reinterpretation of cytogenetic studies in *C*. *furva s*.*l*. [22] shows that *C*. *lucennoiberica* and *C*. *furva s*.*s*. have the same diploid chromosome number (2*n* = 60) with the only exception of an individual of *C*. *furva s*.*s*. which displays an irregular chromosome number of 2*n* = 61.

### Threats and conservation of the species

The highly specific niche requirements of both species (*C*. *furva s*.*s*. and *C*. *lucennoiberica*) are the most limiting factors for their conservation, making them sensitive to climate change and habitat destruction (i.e. soil nitrification). *Carex lucennoiberica* is much endangered in Portugal, where only one individual occurs in Serra da Estrela (population C1; Fig 1), the only population in this country. In Spain, the most threatened population occurs in Sierra de Guadarrama (C5; Fig 1), where only seven individuals have been detected after several recent intensive searches. *Carex furva s*.*s*. seems to be also threatened by hybridization with its most closely related congener, *C*. *lucennoiberica*. Hybridization implies a serious threat for endangered species [58] and can affect the fitness of the species by genetic assimilation or outbreeding depression [59] as has been demonstrated in plants and animals [59–61]. In our study case, four out of 15 sampled individuals from the hybrid population in Sierra Nevada (population S2; Fig 1) showed aborted utricles (pers. obs.) which suggest outbreeding depression as the consequence of hybridization between *C*. *lucennoiberica* and *C*. *furva s*.*s*., which is an additional potential threat for the future conservation of *C*. *furva s*.*s*. Only the populations of *C*. *lucennoiberica* in Sierra de Guadarrama (C5; Fig 1), and *C*. *furva s*.*s*. in the southern Iberian Peninsula (Fig 1) are legally protected by the Spanish government, considered as “sensitive to habitat alteration” in the case of population from Sierra de Guadarrama (C5; Fig 1) and “Vulnerable” in Sierra Nevada. Nevertheless, all existing populations of *C*. *furva s*.*l*. occur in protected natural places, which is indirectly contributing to the conservation of both species.

### Taxonomic treatment

*Carex lucennoiberica* Maguilla & M. Escudero sp. nov. [urn:lsid:ipni.org:names:77158477–1] (Figs 6 and 7, S1 Fig)

Heterotypic synonyms:

= *Carex lagopina* var. *baetica* J. Gay in Ann. Sci. Nat., Bot. ser. 2 11: 181 (1839) (Lectotype: SPAIN: Ulila Lacon mons Sierrae Nevadae altissimus, August 1837. Boissier (s.n.). K000960366, K!, designated here)

            *Carex lagopina* subsp. *baetica* (J. Gay) K. Richt., Pl. Eur. 1: 151 (1890)

            *Carex lachenalii* subsp. *baetica* (J. Gay) Luceño & Muñoz Garm. in Fontqueria 11: 3 (1986)

            *Carex bipartita* subsp. *baetica* (J. Gay) Luceño & Muñoz Garm. in Anales Jard. Bot. Madrid 44: 439 (1987)

Diagnosis–Previously considered within the range of morphological variability of *C*. *furva* Webb. Differs from *C*. *furva s*.*s*. by the usually ovoid or shortly oblong, light brown inflorescence (instead of capitate—aggregated and rounded shape—and dark brown), its utricle beaks nearly appressed to the spike (instead spreading and prominent beaks in the outline of the inflorescence), utricles prominently veined (instead faintly veined), longer culms usually procumbent at maturity (instead shorter and erect), glumaceous or foliose lowest bract of the inflorescence (instead always glumaceous), glumes equal or slightly shorter than utricles (instead shorter than utricles), and utricles slightly smaller, usually the lower ones erect, rarely spreading (instead always spreading). See Table 2 for a detailed comparison of both species.

**Table 2:** Diagnosis characters distinguishing *C*. *lucennoiberica* from its relative *C*. *furva s*.*s*.

	*C*. *furva s*.*s*.	*C*. *lucennoiberica*
**Culm**	2.1–8.6 (10.9) cm	3.7–23.8 (29.2) cm
**Lower bract of the inflorescence**	Glumaceous	Linear or setaceous, sometimes glumaceous
**Inflorescence**	Usually capitate——aggregated and rounded shape—, with utricle beaks clearly prominent in the outline, dark brown	Ovoid or shortly oblong, rarely capitate, with utricle beaks appressed to the spike, not prominent in the outline, light brown
**Female glume**	Much shorter than utricles	As long as or shorter than utricles
**Utricle**	(1.94) 2.15–2.66 mm length, faintly veined, erect to erect-spreading, the lower usually spreading	1.48–2.37 mm length, prominently veined, the lower rarely spreading

Type–SPAIN: Ávila, Sierra de Béjar, arroyo Malillo. 2300 m.a.s.l. Chionophylous species-rich *Nardus* grasslands, with *Nardus stricta*. 07 August 2010. M. Luceño (21ML10), P. Jiménez-Mejías & M. Guzmán. (Holotype, UPOS-4319; isotypes, K, MA, MOR, UPOS).

Description–Rhizome lax or densely cespitose. Culms 3.7–23.8 (29.2) cm × 0.48–1.18 (1.42) mm, trigonous, with acute angles, smooth or slightly scabrid in the upper part, usually procumbent at maturity. Leaves 1.6–7.9 (13.8) cm × 0.64–2.57 mm, shorter than the culms, the longest ones reaching the inflorescences, rarely surpassing it, flat except at the apex, where it becomes trigonous, amphistomatic, smooth, antrorsely scabrid at the apex; ligule 0.26–1.28 (3.1) mm, usually as wide as the leaf blade, obtuse, rounded or emarginated; basal sheaths with blade absent, from entire to fibrous, brown. Lowest bract often linear or setaceous, with widened base having scarious margins, sometimes glumaceous, shorter than inflorescence, up to half its length, antrorsely scabrid on margins. Inflorescence 5.1–15 × 3–9.3 (9.7) mm, ovoid or shortly oblong, rarely capitate—aggregated and rounded shape—, usually light brown, consisting of 3–8 gynecandrous spikes 2.1–7.1 × 1.2–4.1 mm, ovoid to elliptic, erect to erect-spreading, overlapping, sometimes the lowermost distant, erect-spreading. Male glumes ovoid-oblong, often with nerve prolonged in a mucro, sometimes the nerve scabrid; female glumes (0.92) 1.08–2 × 0.64–1.56 mm, as long as or shorter than the utricles, ovoid, with apex variable, often acute or subacute, 1-nerved, reddish-brown, sometimes with scarious-hyaline margins up to 1.52 mm wide. Utricles 1.48–2.37 mm × (0.56) 0.74–1.16 (1.26) mm, plano-convex to slightly biconvex, usually ellipsoid, prominently veined, erect to erect-spreading, the lower rarely spreading, greenish to brown at maturity, gradually attenuated into a beak (0.12) 0.2–0.58 (0.62) mm, truncate to slightly and irregularly bidentate, sometimes with a suture prolonging up to 0.6 (0.7) mm on the abaxial side, sometimes slightly curved at maturity, smooth or rarely with 1 (2) prickles. Achenes (0.98) 1.08–1.48 mm × 0.62–1.04 mm, biconvex or plano-convex, ± elliptical, with a persistent style base shortly cylindrical. 2*n* = 60 [22].

Distribution and habitat–Endemic to high mountains of the Iberian Peninsula: Sierra Segundera, Sierra del Cornón, Fuentes Carrionas (Curavacas), Sierra de Gredos, Sierra de Guadarrama and Sierra Nevada in Spain, and Serra da Estrela in Portugal. Provinces of Ávila, Cáceres, Granada, León, Madrid, Oviedo, Orense, Palencia, Salamanca, Santander, and Zamora in Spain, Beira Alta in Portugal. Inhabiting wet meadows, bogs, snowbeds, streams and lakes border of siliceous mountains. 1800–3200 *m*.*a*.*s*.*l*. Associates include *Calluna vulgaris* (L.) Hull, *Carex nigra* (L.) Reichard, *Erica tetralix* L., *Mucizonia sedoides* (DC.) D.A.Webb, *Nardus stricta* L., *Omalotheca supina* (L.) DC., *Oreochloa elegans* Sennen, *Plantago alpina* L., *Sedum candollei* Boreau, *Spergularia capillacea* (Kindb. & Lange) Willk. and *Trichophorum cespitosum* (L.) Hartm.

Phenology–Flowering and fructification occur from June to September.

Etymology–The specific epithet—*lucennoiberica—*is an acronym of two words: “lucennoi” and “iberica”. The first word—“lucennoi”—honors prof. Dr. Modesto Luceño (born in 1955). He is a Spanish caricologist who leads a research group at the Universidad Pablo de Olavide (Seville, Spain), focusing on the study of the evolution and systematic of the genus *Carex* (Cyperaceae). He is one of the authors of the most comprehensive taxonomic treatment for the whole Cyperaceae family for the Iberian Peninsula. He was the first who detected and published the presence of morphological variability within *C*. *furva s*.*l*. although the presence of intermediate individuals in Sierra Nevada prevented him to describe a new species. The second word—“iberica”—describes the distribution of the species endemic to the Iberian Peninsula (Spain and Portugal).

Nomenclatural note–*Carex lagopina* Wahlenb. var. *baetica* J.Gay was described in 1839 [23]. Because *Carex baetica Auersw*. *ex Willk*. was also already described in 1948, we cannot just combine the previously described variety at the rank of species.

Additional specimens examined (Paratypes)–**PORTUGAL: Beira Alta**. Serra da Estrela, Alto das Salgadeiras. 1850–1900 m.a.s.l. 07-September-1986. M. Luceño (1604bis PV) et al. MA-314898, MA-342288; Serra da Estrela, Alto das Salgadeiras. 17-July-2012. A. Silva (s.n.). UPOS-5015; **SPAIN: Ávila**. Sierra de Béjar, entre La Covatilla y Cuerda del Calvitero. 2250 m.a.s.l. 20-July-2011. M. Luceño (11ML11). UPOS-5052; Sierra de Béjar, Ceja del Calvitero. 2300 m.a.s.l. 07-July-2010. M. Luceño (26ML10) et al. UPOS-4324; Sierra de Béjar, Ceja del Calvitero. 2300 m.a.s.l. 28-July-1982. E. Rico (s.n.). MA-248644; Sierra de Béjar, Ceja del Calvitero. 2300 m.a.s.l. 28-July-1982. E. Rico (300). SEV-92694; Sierra de Béjar, Lagunas del Trampal. 27-September-1979. Amich (s.n.) et al. MA-236946; Sierra de Candelario, La Ceja. 26-July-1989. S. Rivas-Martínez (217) et al. MA-616279; Sierra del Barco, Alto de Castilfrío. 2184 m.a.s.l. 15-July-2012. E. Maguilla (23EMS12) et al. UPOS-5039; Sierra del Barco, cresta de la Covacha del Lósar. 2300 m.a.s.l. 8-July-1984. M. Luceño (s.n.). MA-267018; Sierra del Barco, cresta de la Covacha del Lósar, 2325 m.a.s.l. 08-July-1984. M. Luceño (s.n.). MA-342292; Sierra del Barco, cresta de la Covacha del Lósar. 08-July-1984. M. Luceño (s.n.). MA-283921; Sierra de Gredos, base de la portilla de Los Cobardes. 2435 m.a.s.l. 26-September-2004. M. Luceño (1804ML) & L. E. Vendrell. UPOS-5050; Sierra de Gredos, base del Ameal de Pablo. 2410 m.a.s.l. 29-July-1985. M. Luceño (s.n.) et al. MA-293877; Sierra de Gredos, entre la portilla del Crampón y elAlmanzor. 2400 m.a.s.l. 31-August-1984. M. Luceño (s.n.). MA-292878; Sierra de Gredos, entre El Venteadero y La Galana. 2476 m.a.s.l. 14-July-2012. E. Maguilla (15EMS12) & M. Luceño. UPOS-5037; Sierra de Gredos, circo de Cinco Lagunas. 28-July-1985. M. Luceño (s.n.) et al. MA-342291, MA-406377; Sierra de Gredos, circo de Cinco Lagunas. 2115 m.a.s.l. 29-July-1985. M. Luceño (s.n.) et al. MA-291852; Sierra de Gredos, circo de Cinco Lagunas. 2120 m.a.s.l. July-1985. M. Luceño (s.n.) et al. MA-292876; Sierra de Gredos, circo de Cinco Lagunas. 2120 m.a.s.l. 29-July-1985. M. Luceño (s.n.) et al. MA-292875; Sierra de Gredos, portilla del Ameal. 2400 m.a.s.l. 8-July-1989. S. Castroviejo (10794SC) et al. MA-480018; Sierra de Gredos, laguna del Gutre lagoon. 2310 m.a.s.l. 17-August-2014. M. Luceño (206ML14BIS). UPOS-6231; Sierra de Gredos, El Venteadero. 2500 m.a.s.l. 29-July-1985. M. Luceño (s.n.) et al. MA-292881; Sierra de Gredos, El Venteadero. 2500 m.a.s.l. 31-August-1984. M. Luceño (s.n.). MA-292880, MA-342294; Sierra de Gredos, El Venteadero. 2518 m.a.s.l. 14-July-2012. E. Maguilla (16EMS12) & M. Luceño UPOS-5038; Sierra de Gredos, La Mira, cara norte. 2221 m.a.s.l. 28-June-2015. M. Luceño (473ML15) & S. Guerra-Cárdenas. UPOS-6575; Sierra de Gredos, laguna Grande. 1900 m.a.s.l. 28-June-1987. Gómez-Manzaneque (PV2319) et al. MA-406542; Sierra de Gredos, garganta de Los Conventos. 2000 m.a.s.l. 19-August-2014. M. Luceño (243ML14). UPOS-6230; Sierra de Gredos, fuente Los Serranos. 2350 m.a.s.l. 31-August-1984. M. Luceño (s.n.). MA-292879, MA-406378; Sierra de Gredos, Puerto Castilla. 1-July-1999. P. Vargas (178PV99). MA-757012; Sierra de Gredos, Puerto Castilla, laguna del Barco. 28-July-1984. E. Rico (s.n.) & J. Sánchez-Rodríguez. MA-317737; **Cáceres**. Tornavacas, portilla de Jaranda. 27-July-1985. X. Giráldez (s.n.) & E. Rico. MA-317738; **Cantabria**. Vega de Liébana, cerca de Peña Prieta. 2100 m.a.s.l. 14-August-1987. C. Aedo (s.n.). MA-622677; **Granada.** Sierra Nevada, Lagunillos de la Virgen. 2960 m.a.s.l. 25-August-1985. M. Luceño (s.n.) et al. MA-292870; Sierra Nevada, Mulhacén septentrional. 2400–2900 m.a.s.l. August-1834. Boissier (s.n.). K-s.n; **Madrid.** Sierra de Guadarrama, Risco de los Pájaros. 2323 m.a.s.l. 22-August-2013. E. Maguilla (35EMS13) & T. Villaverde. UPOS-5141; **Orense**. Sierra Segundera, entre Peña Trevinca y el pico Jancional. 2085 m.a.s.l. 24-August-2013. E. Maguilla (39EMS13). UPOS-5117; **Oviedo.** Concejo de Somiedo, El Cornón. 2000 m.a.s.l. 26-August-1985. I. Aizpuru (7237). MA-292886, MA-342295; Concejo de Somiedo, El Cornón. 2012 m.a.s.l. 23-August-2013. E. Maguilla (36EMS13) & T. Villaverde. UPOS-5136; Concejo de Somiedo, El Cornón. 2100 m.a.s.l. 26-August-1985. I. Aizpuru (3408.85). MA-823941; Concejo de Somiedo, El Cornón, cerca de Villar de Vildas. 2000 m.a.s.l. 26-August-1985. C. Aedo (s.n.). MA-622678; **Palencia**. Curavacas, lagunas de Fuentes Carrionas. 2200 m.a.s.l. 11-August-2005. C. Aedo (12234). MA-732663; Curavacas, cara norte. 1800–2300 m.a.s.l. 24-August-1986. Argüelles (s.n.) et al. MA-308707, MA-342289; Curavacas, cara norte. 2000 m.a.s.l. 30-August-2007. S. Martín-Bravo (172SMB07) & P. Jiménez-Mejías. UPOS-5054; Curavacas, sender desde lo alto del Curavacas a El Pozo. 2400 m.a.s.l. 15-August-1985. M. Luceño (s.n.) et al. MA-292874, MA-342296; **Salamanca**. Sierra de Candelario, El Calvitero. 2300 m.a.s.l. 30-June-1985. M. Luceño (s.n.). MA-342293; Sierra de Candelario, El Calvitero, cara noroeste. 2300 m.a.s.l. 18-July-1980. E. Valdés-Bermejo (5809EV) et al. MA-292887; **Zamora**. Porto, Moncalvo. 1980 m.a.s.l. 27-July-2002. P. Bariego (PB-2363) & E. Rico. MA-793227; Porto, Moncalvo. 2000 m.a.s.l. 30-July-2002. P. Bariego (PB-841) & E. Rico. MA-793228.

*Carex furva* Webb, Iter Hispan.: 5 (1838). (Fig 5 and S2 Fig)

Homotypic synonyms:

*≡ Carex lagopina* var. *furva* (Webb) Webb, Otia Hispan.: 46 (1839).

            *Carex lachenalii* var. *furva* (Webb) C.Vicioso, Bol. Inst. Forest. Invest. Exp. 30(79): 67 (1959).

            *Carex lachenalii* subsp. *furva* (Webb) Malag., Sin. Fl. Ibér. 7: 142 (1980), *comb*. *inval*.

Type–SPAIN: Granada, Sierra Nevada, *in Baeticae montibus altioribus*. April 1838. Webb (s.n). (Lectotype designated by H. Toivonen in Ann. Bot. Fenn. 16:16 (1979), K000960368, K!; Isolectotype, FI012265, FI image!).

Description–Rhizome lax or densely cespitose. Culms 2.1–8.6 (10.9) cm × 0.5–1.26 (1.62) mm, trigonous, with acute angles, smooth or slightly scabrid in the upper part, erect at maturity. Leaves 0.8–5.6 cm × 0.64–2.3 mm, usually shorter than the culms, sometimes lightly longer, flat except at the apex, where it becomes trigonous, amphistomatic, smooth, antrorsely scabrid at the apex; ligule 0.32–0.98 mm, usually as wide as the leaf blade, obtuse, rounded or emarginated; basal sheaths with blade absent, entire or fibrous, brown. Lowest bract glumaceous, shorter than inflorescence, with scarious and scabrid margin at the apex. Inflorescence (4.3) 4.8–9.4 (11) × 3.7–9.4 (11) mm, usually capitate——aggregated and rounded shape—, sometimes ovoid to shortly oblong, dark brown, consisting of 3–5 gynecandrous spikes of 3–6.1 (6.9) × 1.5–5.1 mm, ovoid to elliptic, erect to erect-spreading, overlapping, the lowermost spreading or, more rarely, erect-spreading. Male glumes ovoid-oblong, with short nerve; female glumes (1.16) 1.34–2 × 0.92–1.28 (1.32) mm, much shorter than the utricles, usually ovate, with apex variable, 1-nerved, with the nerve sometimes prolonged in a short mucro, reddish-brown, sometimes with scarious-hyaline margins up to 0.98 mm wide. Utricles (1.94) 2.15–2.66 mm × 0.68–1.08 mm, plano-convex to slightly biconvex, usually ellipsoid, faintly veined, erect to erect-spreading, the lower usually spreading, brown to dark brown at maturity in areas protruding from the glumes, rarely greenish at maturity, gradually attenuated into a beak of (0.4) 0.48–0.72 (0.82) mm, truncate to slightly and irregularly bidentate, sometimes with a suture prolongued up to 0.74 mm on the abaxial side, sometimes slightly curved at maturity, smooth. Achenes (1.1) 1.38–1.58 (1.66) mm × (0.64) 0.76–0.92 (0.98) mm, biconvex or plano-convex, ellipsoid, with a persistent style base shortly cylindrical. 2*n* = 60, 61 [22].

Distribution and habitat–Endemic to Sierra Nevada, Granada province, Spain. Occurring in wet meadows, bogs, snowbeds, streams and lakes border of siliceous mountains. 2700–3200 m.a.s.l. Associates include *Agrostis canina* subsp. *granatensis* Romero Garcia, Blanca & Morales, *Agrostis nevadensis* Boiss., *Carex lepidocarpa* subsp. *nevadensis* (Boiss. & Reut.) Luceño, *Carex nigra* (L.) Reichard., *Euphrasia willkommii* Freyn, *Festuca frigida* Grossh., *Gentiana boryi* Boiss., *Gentiana pneumonanthe* subsp. *depressa* (Boiss.) Malag., Asensi, Molero Mesa & F. Valle, *Leontodon microcephalus* Boiss., *Nardus stricta* L., *Ranunculus angustifolius* subsp. *alismoides* (Bory) Malag., *Sagina nevadensis* Boiss. & Reut., *Veronica nevadensis* (Pau) Pau, and *Viola palustris* L.

Phenology–Flowering and fructification from (June) July to August (October).

Additional specimens examined–**Granada.** Sierra Nevada, Barranco de Trevélez. S. de R. Clemente (s.n.). MA-18516; Sierra Nevada, Borreguiles. 2800 *m*.*a*.*s*.*l*. 18-July-1976. A. Barra et al. (854bis EV). MA-437957; Sierra Nevada, Borreguiles. 3142.5 *m*.*a*.*s*.*l*. 12-August-2011. A. Jiménez-Bonilla (1AJB11). UPOS-5053; Sierra Nevada, corral del Veleta. 3120 *m*.*a*.*s*.*l*. 23-August-1985. M. Luceño et al. (s.n.). MA-292884, MA-342298; Sierra Nevada, corral de Valdeinfiernos. 2860 *m*.*a*.*s*.*l*. 31-August-1985. R. Vogt (s.n.). MA-292871; Sierra Nevada, Hoya de la Mora. 27-July-1967. A. Segura-Zubizarreta (8749). MA-293261; Sierra Nevada, *in Baeticae montibus altioribus*. April 1838. Webb (s.n) K-s.n., FI-s.n. (TYPE); Sierra Nevada, laguna de Aguas Verdes. 3085 *m*.*a*.*s*.*l*. 19-August-2006. P. Jiménez-Mejías & M. Escudero (158PJM06). UPOS-3832; Sierra Nevada, laguna de Aguas Verdes. 3098–3126 *m*.*a*.*s*.*l*. 08-August-2013. E. Maguilla & J. M. G. Cobos (31EMS13). UPOS-5132; Sierra Nevada, laguna de la Mosca. 3000 *m*.*a*.*s*.*l*. 02-October-1975. F. Casas & García-Guardia (975). MA-394000; Sierra Nevada, laguna de la Mosca. 3000 *m*.*a*.*s*.*l*. 31-August-1985. R. Vogt (s.n.). MA-292873; Sierra Nevada, laguna de las Yeguas. 26-August-1969. B. Lippert & W. Lippert (10035). Sierra Nevada, laguna de las Yeguas. 2750 *m*.*a*.*s*.*l*. 22-August-1985. M. Luceño et al. (691PV). MA-342299, M-0177641; Sierra Nevada, laguna de las Yeguas. 2830 *m*.*a*.*s*.*l*. 27-June-1980. J. A. Devesa et al. (1708/80). SEV-161471; Sierra Nevada, laguna de las Yeguas. 2900 *m*.*a*.*s*.*l*. 02-July-1986. C. Aedo (s.n.). MA-622680; Sierra Nevada, laguna de las Yeguas. 2985 *m*.*a*.*s*.*l*. 22-August-1985. M. Luceño et al. (s.n.). MA-292883; Sierra Nevada, lagunas y arroyos tributarios al embalse de las Yeguas. 2860 *m*.*a*.*s*.*l*. 19-August-2006. P. Jiménez-Mejías & M. Escudero (161PJM06). UPOS-3833; Sierra Nevada, laguna de Río Seco. 3040 *m*.*a*.*s*.*l*. 22-August-1985. M. Luceño et al. (s.n.). MA-342300, MA-292882; Sierra Nevada, Lagunillos de la Virgen. 2960 *m*.*a*.*s*.*l*. 25-August-1985. M. Luceño et al. (s.n.). MA-292872, MA-292870, MA-292869, MA-342290, MA-342297, MA-292885; Sierra Nevada, Siete Lagunas. 2940 *m*.*a*.*s*.*l*. 30-July-1997. J. M. López-Nieto (s.n.). MA-873097; Sierra Nevada, valle de Lanjarón, 07-August-1930. L. Ceballos & C. Vicioso (s.n.). MA-17094; Sierra Nevada, Veleta. 02-July-1965. D. M. Moore (1201). BM-s.n; Sierra Nevada, Veleta. 31-July-1876. M. Minkler (s.n.). M-0177640; Sierra Nevada, Veleta. 29-August-1966. R. M. Harley & A. M. Harley (1055). BM-s.n.

## Conclusions

The taxonomy of the genus *Carex* has been defined to be sometimes problematic due to hybridization of species [13,14] preventing the finding of morphological discontinuities between taxa that remain cryptic. Even in a group—*Carex* section *Glareosae*—where hybrid specimens are in most cases sterile [20,26,27] and hybrid speciation seems to be not relevant from an evolutionary point of view [19,20], hybridization can hinder the detection and characterization of incipient species. Combination of morphological and molecular data with differential treatment of hybrid populations has allowed the description of a new cryptic species endemic to the Iberian Peninsula, *C*. *lucennoiberica*.

## Supporting Information

S1 Table Measurements of morphological variables. *A priori* and *a posteriori* identifications based on our results are shown, as well as label information and herbarium identifier, and population name as in Fig 1.
(XLS)

S1 Fig Detailed pictures of *Carex lucennoiberica* Maguilla & M. Escudero. SPAIN: Madrid, Sierra de Guadarrama, Rascafría, Sierra de Guadarrama Nacional Park. 22 August 2013. E. Maguilla (35EMS13(5)) & T. Villaverde. UPOS-5141. (A) General aspect—scale bar = 1 cm—; (B) Inflorescence; (C) utricle, abaxial view; (D) utricle, adaxial view. Scale bar in B, C and D = 1 mm.
(TIF)

S2 Fig Detailed pictures of *Carex furva* Webb. SPAIN: Granada, Sierra Nevada, Capileira, Sierra Nevada National Park. 08 August 2013. E. Maguilla (31EMS13(15)) & J. M. G. Cobos. UPOS-5132. (A) General aspect—scale bar = 1 cm—; (B) Inflorescence; (C) utricle, abaxial view; (D) utricle, adaxial view. Scale bar in B, C and D = 1 mm.
(TIF)

S1 File NCBI Genbank accession numbers of the samples included in phylogenetic analyses. Locality of collection and accession numbers are shown (ETS, ITS, G3PDH, *mat*K).
(DOCX)

S2 File Results from Kaiser-Meyer-Olkin (KMO), Bartlett’s tests and principal component analyses (PCA). Results derived from the analyses implemented in IBM SPSS Statistics v.20 (IBM Inc., Chicago, IL, USA) using morphological variables measured in *Carex furva s*.*l*. (A) Results of the analyses including all 21 measured variables and the hybrid population. (B) Results of the PCA analysis including nine selected variables and the hybrid population. (C) Results including all 21 variables and excluding the hybrid population. (D) Results of the analysis using 11 selected variables and excluding the hybrid population.
(DOC)

S3 File Discriminant function analysis (DFA) results. Results derived from the analyses implemented in IBM SPSS Statistics v.20 (IBM Inc., Chicago, IL, USA) using morphological variables measured in *Carex furva s*.*l*. Variables included: CLMW, INFL, INFW, USPIKA, SLSPIKA, LSPIKA, SPIKL, SPIKW, PERL, PERW, PERBKL, PERMWD, PERSTL, PSCLL, PSCLW, MINHYAL, MAXHYAL, ACHL, ACHW, SPKN and PERIGTHN.
(DOCX)

S4 File Test of normality, ANOVA and Mann-Whitney U test results. Results derived from the analyses implemented in IBM SPSS Statistics v.20 (IBM Inc., Chicago, IL, USA) using 21 morphological variables: CLMW, INFL, INFW, USPIKA, SLSPIKA, LSPIKA, SPIKL, SPIKW, PERL, PERW, PERBKL, PERMWD, PERSTL, PSCLL, PSCLW, MINHYAL, MAXHYAL, ACHL, ACHW, SPKN and PERIGTHN.
(DOCX)

## References

[1] Judd WS, Campbell CS, Kellogg EA, Stevens PF, Donoghe MJ. Plant systematics: A phylogenetic approach, 3rd edn Sinauer Associates, Inc, Sunderland. Kluge; 2007.

[2] Global Carex Group. Making *Carex* monophyletic (Cyperaceae, tribe *Cariceae*): a new broader circumscription. Bot J Linn Soc. 2015; 179: 1–42.

[3] Govaerts R, Simpson DA, Bruhl J, Egorova TV, Goetghebeur P, Wilson K. World checklist of Cyperaceae Sedges. In: Royal Botanical Gardens, Kew; 2007.

[4] Escudero M, Hipp AL, Waterway MJ, Valente LM. Diversification rates and chromosome evolution in the most diverse angiosperm genus of the temperate zone (*Carex*, Cyperaceae). Mol Phylogenet Evol. 2012; 63: 650–655. 10.1016/j.ympev.2012.02.005 22366369

[5] Escudero M, Hipp AL. Shifts in diversification rates and clade ages explain species richness in higher-level sedge taxa (Cyperaceae). Am J Bot. 2013; 100: 2403–2411. 10.3732/ajb.1300162 24249788

[6] Waterway MJ, Hoshino T, Masaki T. Phylogeny, species richness, and ecological specialization in Cyperaceae tribe *Cariceae*. Bot Rev. 2009; 75: 138–159.

[7] Roalson EH, Columbus JT, Friar EA. Phylogenetic relationships in *Cariceae* (Cyperaceae) based on ITS (nrDNA) and *trn*T-L-F (cpDNA) region sequences: Assessment of subgeneric and sectional relationships in *Carex* with emphasis on section *Acrocystis*. Syst Bot. 2001; 26: 318–341.

[8] Maguilla E, Escudero M, Waterway MJ, Hipp AL, Luceño M. Phylogeny, systematics, and trait evolution of *Carex* section *Glareosae*. Am J Bot. 2015; 102: 1128–1144. 10.3732/ajb.1500169 26199369

[9] Escudero M, Luceño M. Systematics and evolution of *Carex* sects. *Spirostachyae* and *Elatae* (Cyperaceae). Plant Syst Evol. 2009; 279: 163–189.

[10] Jiménez-Mejías P, Martín-Bravo S, Luceño M. Systematics and taxonomy of *Carex* sect. *Ceratocystis* (Cyperaceae) in Europe: A molecular and cytogenetic approach. Syst Bot. 2012; 37: 382–398.

[11] Global Carex Group. Megaphylogenetic specimen-level approaches to the *Carex* (Cyperaceae) phylogeny using ITS, ETS, and *mat*K sequences: implications for classification. Syst Bot. 2016; 41: 500–518.

[12] Kukkonen I, Toivonen H. Taxonomy of wetland carices. Aquat Bot. 1988; 30: 5–22.

[13] Cayouette J, Catling P. Hybridization in the genus *Carex* with special reference to North America. Bot Rev. 1992; 58: 351–438.

[14] Escudero M, Eaton DAR, Hahn M, Hipp AL. Genotyping-by-sequencing as a tool to infer phylogeny and ancestral hybridization: A case study in *Carex* (Cyperaceae). Mol Phylogenet Evol. 2014; 79: 359–367. 10.1016/j.ympev.2014.06.026 25010772

[15] Barton NH, Hewitt GM. Analysis of hybrid Zones. Annu Rev Ecol Syst. 1985; 16: 113–148.

[16] Dépraz A, Hausser J, Pfenninger M. A species delimitation approach in the *Trochulus sericeus/hispidus* complex reveals two cryptic species within a sharp contact zone. BMC Evol Biol. 2009; 9: 171 10.1186/1471-2148-9-171 19622149 PMC2724411

[17] Lemmon EM, Lemmon AR, Collins JT, Lee-Yaw JA, Cannatella DC. Phylogeny-based delimitation of species boundaries and contact zones in the trilling chorus frogs (Pseudacris). Mol Phylogenet Evol. 2007; 44: 1068–1082. 10.1016/j.ympev.2007.04.010 17562372

[18] Kauserud H, Svegården IB, Decock C, Hallenberg N. Hybridization among cryptic species of the cellar fungus *Coniophora puteana* (Basidiomycota). Mol Ecol. 2007; 16: 389–399. 10.1111/j.1365-294X.2006.03129.x 17217352

[19] Egorova TV. The sedges (*Carex* L.) of Russia and adjacent states (Within the limits of the former USSR). St. Louis: St. Petersburg State Chemical-Pharmaceutical Academy, St. Petersburg and Missouri Botanical Garden; 1999.

[20] Toivonen H. *Carex* L. sect. *Glareosae* G.Don (Cyperaceae). Flora of North America, north of Mexico. Flora of North America Editorial Committee. Oxford: Oxford University Press; 2002 pp. 311–321.

[21] Kükenthal G. Cyperaceae-Caricoidae In: Engler A, editor. Das Pflanzenreich. Leipzig: W. Englemann; 1909 pp. 20–38.

[22] Luceño M. Estudios en el género *Carex*. I. Sección *Canescentes* (Fries) Christ.: *C*. *furva* Webb y *C*. *lachenalii* Schkuhr. An Jard Bot Madr. 1986; 42: 427–440.

[23] Gay J, Ann. Sci. Nat. Bot. sér. 2, 11. París 1839 pp. 179–181.

[24] Latch EK, Harveson LA, King JS, Hobson MD, Rhodes OE. Assessing hybridization in wildlife populations using molecular markers: a case study in wild turkeys. J Wildl Manage. 2006; 70: 485–492.

[25] Oliveira R, Godinho R, Randi E, Alves PC. Hybridization versus conservation: are domestic cats threatening the genetic integrity of wildcats (*Felis silvestris silvestris*) in Iberian Peninsula? Philos Trans R Soc Lond B Biol Sci. 2008; 363: 2953–2961. 10.1098/rstb.2008.0052 18522917 PMC2606743

[26] Toivonen H. *Carex canescens* × *mackenziei*. A comparative study of two *Carex* species and their spontaneous hybrid. Ann Bot Fenn. 1980; 17: 91–123.

[27] Toivonen H. Spontaneous *Carex* hybrids of *Heleonastes* and related sections in Fennoscandia. Acta Bot Fenn. 1981; 116: 1–51.

[28] Luceño M. *Carex* L In: Castroviejo S, Luceño M, Galan A, Jiménez-Mejías P, Cabezas F, Medina L, editors. Flora iberica. Madrid: Real Jardín Botánico, CSIC; 2008 pp. 146–151.

[29] Luceño M, Maguilla E, Escudero M, Silva A, Guerra-Cárdenas S, Hilpold A, et al Notas de la familia Ciperáceas en la Península Ibérica. Acta Bot Malacit. 2015; 40: 217–221.

[30] Thiers B. Index Herbariorum: A global directory of public herbaria and associated staff; 2015 New York Botanical Garden's Virtual Herbarium http://sweetgum.nybg.org/ih/

[31] Webb PB. Iter Hispaniense. 5. Paris. 1838.

[32] Jiménez-Mejías P, Luceño M, Martín-Bravo S. Species boundaries within the southwest Old World populations of the *Carex flava* Group (Cyperaceae). Syst Bot. 2014; 39: 117–131.

[33] Valcárcel V, Vargas P. Quantitative morphology and species delimitation under the general lineage concept: Optimization for *Hedera* (Araliaceae). Am J Bot. 2010; 97: 1555–73. 10.3732/ajb.1000115 21616907

[34] Tabachnick BG, Fidell LS. Using multivariate statistics, 5th ed Allyn and Bacon, Boston, Massachusetts, USA 2007.

[35] Edgar RC. MUSCLE: a multiple sequence alignment method with reduced time and space complexity. BMC Bioinformatics. 2004; 5: 113 10.1186/1471-2105-5-113 15318951 PMC517706

[36] Darriba D, Taboada GL, Doallo R, Posada D. jModelTest 2: more models, new heuristics and parallel computing. Nat Methods. 2012; 9: 772. 10.1038/nmeth.2109 PMC4594756 22847109

[37] Akaike H. A new look at the statistical model identification. IEEE Trans Automat Contr. 1974; 19: 716–723.

[38] Simmons M, Ochoterena H. Gaps as characters in sequence-based phylogenetic analyses. Syst Biol. 2000;49: 369–381. 12118412

[39] Ronquist F, Huelsenbeck JP. MrBayes 3: Bayesian phylogenetic inference under mixed models. Bioinformatics. 2003; 19: 1572–1574. 12912839 10.1093/bioinformatics/btg180

[40] Cronquist A. The evolution and classification of flowering plants, ed. 2. New York. 1988.

[41] Cracraft J. Speciation and its ontology: the empirical consequences of alternative species concepts for understanding patterns and processes of differentiation. In: Otte D, Endler, JA, editors. Speciation and its consequences. Sunderland MA. 1989. pp 28–59.

[42] de Queiroz A. The resurrection of oceanic dispersal in historical biogeography. Trends Ecol Evol. 2005; 20: 68–73. 10.1016/j.tree.2004.11.006 16701345

[43] Dobzhansky T. Genetics and the origin of species, ed. 2. New York. 1941.

[44] Mayr E. The biological meaning of species. Bot J Linn Soc. 1969; 1: 311–320.

[45] Yang H, Wang Q, Bai C, Li X, Liu G. *Carex diaoluoshanica* (*Carex* sect. *Lageniformes*, cyperaceae), a new species from Hainan, China. PLoS One. 2014; 9: 1–6. 10.1371/journal.pone.0097658 PMC4061016 24937208

[46] Yang H, Li X, Wang W, Bai C, Liu G. *Carex jianfengensis* (*Carex* sect. *Rhomboidales*, Cyperaceae), a new species from Hainan, China. PLoS One. 2015; 10: e0136373 10.1371/journal.pone.0136373 26397809 PMC4580456

[47] Leaché AD, Fujita MK. Bayesian species delimitation in West African forest geckos (*Hemidactylus fasciatus*). Proc Biol Sci. 2010; 277: 3071–3077. 10.1098/rspb.2010.0662 20519219 PMC2982061

[48] Bauer AM, Parham JF, Brown RM, Stuart BL, Grismer L, Papenfuss TJ, et al Availability of new Bayesian-delimited gecko names and the importance of character-based species descriptions. Proc R Soc Lond B Biol Sci. 2011; 278: 490–492. 10.1098/rspb.2010.1330 PMC3025678 20961901

[49] Edwards DL, Knowles LL. Species detection and individual assignment in species delimitation: can integrative data increase efficacy? Proc R Soc Lond B Biol Sci. 2014; 281: 20132765. 10.1098/rspb.2013.2765 PMC3896021 24403337

[50] Marino IAM, Riginella E, Cariani A, Tinti F, Farrell ED, Mazzoldi C, et al New molecular tools for the identification of 2 endangered smooth-hound sharks, *Mustelus mustelus* and *Mustelus punctulatus*. J Hered. 2015; 106: 123–130. 10.1093/jhered/esu064 25425673

[51] Watanabe MT, Hensold N, Sano PT. *Syngonanthus androgynus*, a striking new species from South America, its phylogenetic placement and implications for evolution of bisexuality in Eriocaulaceae. PLoS One. 2015; 10: 1–15. 10.1371/journal.pone.0141187 PMC4641623 26559183

[52] Howlader MSA, Nair A, Gopalan S V., Meril?? J. A new species of *Microhyla* (Anura: Microhylidae) from Nilphamari, Bangladesh. PLoS One. 2015; 10: 1–18. 10.1371/journal.pone.0119825 PMC4373918 25806804

[53] Martín-Bravo S, Escudero M, Míguez M, Jiménez-Mejías P, Luceño M. Molecular and morphological evidence for a new species from South Africa: *Carex rainbowii* (Cyperaceae). S Afr J Bot. 2013; 87: 85–91.

[54] Lamer JT, Sass GG, Boone JQ, Arbieva ZH, Green SJ, Epifanio JM. Restriction site-associated DNA sequencing generates high-quality single nucleotide polymorphisms for assessing hybridization between bighead and silver carp in the United States and China. Mol Ecol Resour. 2014; 14: 79–86. 10.1111/1755-0998.12152 23957862

[55] Yang R, Brice B, Jian F, Ryan U. Morphological and molecular characterization of *Isospora manorinae* n. sp. in a yellow-throated miner (*Manorina flavigula wayensis*) (Gould, 1840). Exp Parasitol. 2016; 163: 16–23. 10.1016/j.exppara.2016.01.013 26821297

[56] Martín-Bravo S, Jiménez-Mejías P. Molecular data helps traditional taxonomy: Re-evaluation of *Reseda collina* (Resedaceae), and a new record for Europe. Folia Geobot. 2009; 44: 399–421.

[57] Jiménez-Mejías P, Escudero M, Guerra-Cárdenas S, Lye KA, Luceño M. Taxonomic delimitation and drivers of speciation in the Ibero-North African Carex sect. Phacocystis river-shore group (Cyperaceae). Am J Bot. 2011; 98: 1855–1867. 10.3732/ajb.1100120 22025295

[58] Costedoat C, Pech N, Chappaz R, Gilles A. Novelties in hybrid zones: Crossroads between population genomic and ecological approaches. PLoS One. 2007; 2: e357 10.1371/journal.pone.0000357 17406681 PMC1831490

[59] Ellstrand NC, Elam DR, Ellstrand NC, Elam DR. Population genetic consequences of small population size: Implications for plant conservation. Annu Rev Ecol Syst. 1993; 24: 217–242.

[60] Rhymer JM, Simberloff D. Extinction by hybridization and introgression. Annu Rev Ecol Syst. 1996; 27: 83–109.

[61] Dowling TE, Secor CL. The role of hybridization and introgression in the diversification of animals. Annu Rev Ecol Syst. 1997; 28: 593–619.

[62] Brummitt RK, Pando F, Hollis S, Brummitt NA. World geographical scheme for recording plant distributions, 2 edn Carnegie Mellon University, Pittsburgh: International Working Group on Taxonomic Databases For Plant Sciences, Hunt Institute for Botanical Documentation. 2001.

[63] Jarvis A, Reuter HI, Nelson A, Guevara E. Hole-filled SRTM for the globe Version 4, available from the CGIAR-CSI SRTM 90m Database (http://srtm.csi.cgiar.org). 2008.

