# Correction: Cryptic Species Due to Hybridization: A Combined Approach to Describe a New Species (*Carex*: Cyperaceae)

**DOI:** 10.1371/journal.pone.0172079

**Published:** 2017-02-09

**Authors:** Enrique Maguilla, Marcial Escudero

There is an error in the designation of the Type specimen under the subheading “Taxonomic Treatment” in the Discussion section. The designation should read: Type–SPAIN: Ávila, Sierra de Béjar, arroyo Malillo. 2300 m.a.s.l. Chionophylous species-rich *Nardus* grasslands, with *Nardus stricta*. 07 August 2010. M. Luceño (21ML10), P. Jiménez-Mejías & M. González. (Holotype, UPOS-4319; isotypes, K, MA, MOR, UPOS).
