# Supplementary figures and images for: Cryptic Species Due to Hybridization: A Combined Approach to Describe a New Species (Carex: Cyperaceae)
Source: PLoS One. 2016 Dec 14;11(12):e0166949. doi: 10.1371/journal.pone.0166949 (PMC5156347; doi:10.1371/journal.pone.0166949)

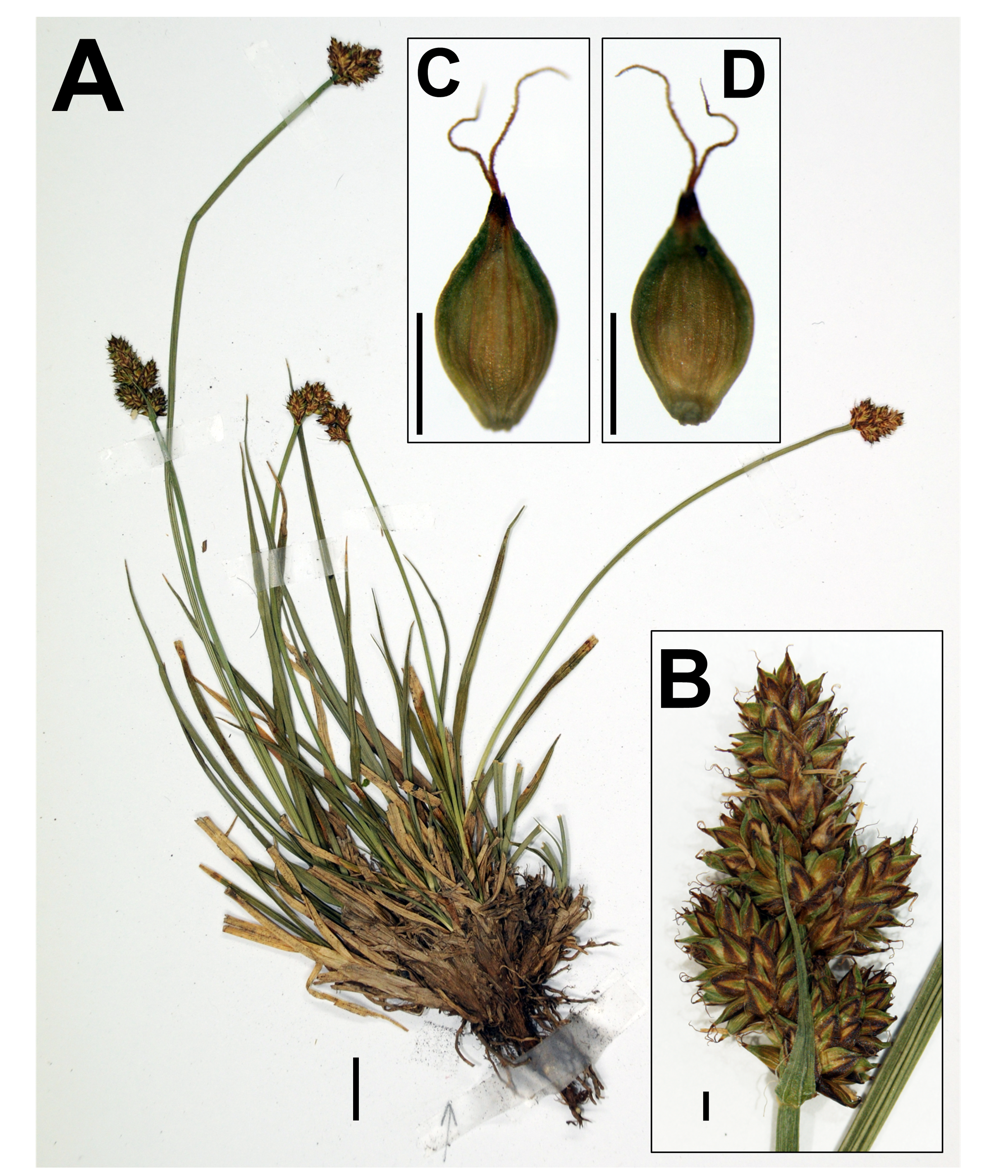

Supplement: S1 Fig — SPAIN: Madrid, Sierra de Guadarrama, Rascafría, Sierra de Guadarrama Nacional Park. 22 August 2013. E. Maguilla (35EMS13(5)) & T. Villaverde. UPOS-5141. (A) General aspect—scale bar = 1 cm—; (B) Inflorescence; (C) utricle, abaxial view; (D) utricle, adaxial view. Scale bar in B, C and D = 1 mm. (TIF) [file pone.0166949.s002.tif]

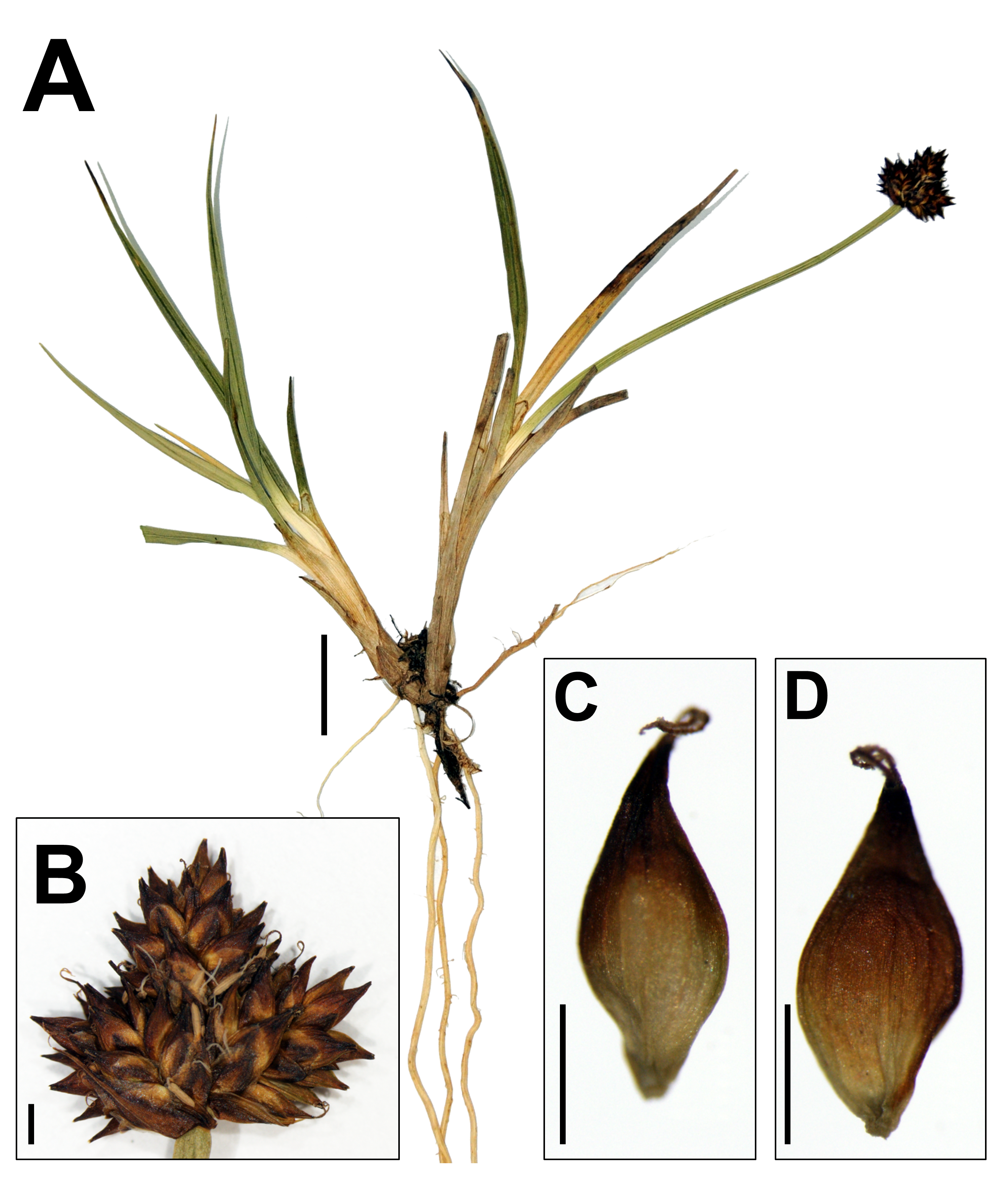

Supplement: S2 Fig — SPAIN: Granada, Sierra Nevada, Capileira, Sierra Nevada National Park. 08 August 2013. E. Maguilla (31EMS13(15)) & J. M. G. Cobos. UPOS-5132. (A) General aspect—scale bar = 1 cm—; (B) Inflorescence; (C) utricle, abaxial view; (D) utricle, adaxial view. Scale bar in B, C and D = 1 mm. (TIF) [file pone.0166949.s003.tif]
