# Supplementary material for: Cryptic Species Due to Hybridization: A Combined Approach to Describe a New Species (Carex: Cyperaceae)
Source: PLoS One. 2016 Dec 14;11(12):e0166949. doi: 10.1371/journal.pone.0166949 (PMC5156347; doi:10.1371/journal.pone.0166949)
Supplement: S1 File — Locality of collection and accession numbers are shown (ETS, ITS, G3PDH, matK). (DOCX) [file pone.0166949.s004.docx]

**Supporting Information**

**S1 File. NCBI Genbank accession numbers of the samples included in phylogenetic analyses.**

*Carex furva sensu lato*:

***C. furva:*** Portugal, Covilhã, Serra da Estrela (KP980336, KP980528, KP979968, KP980138). Spain_1, Ávila, Gredos mountain range (KP980314, KP980504, KP979949, KP980120). Spain_2, Ávila, Béjar mountain range (KP980226, KP980413, KP979869, KP980043). Spain_3, Ávila, Béjar mountain range (KP980289, KP980478, KP979925, KP980101). Spain_4, Granada, Sierra Nevada (KP980274, KP980462, KP979912, KP980087). Spain_5, Granada, Sierra Nevada (KP980331, KP980522, KP979962, KP980132). Spain_6, Oviedo, Somiedo mountain range (KP980304, KP980494, KP979939, KP980112). Spain_7, Palencia, Curavacas Mountain (KP980247, KP980434, KP979887, KP980064).

Species in the sister clade to *Carex furva*:

***C. billingsii:*** Canada, Ontario, Perth County (KP980267, KP980455, KP979905, KP980160); ***C. brunnescens:*** Sweden, Jämtland, Åre s:n (KP980297, KP980487, KP979934, KP980109); ***C. diastena:*** Russian Federation, Sakhalin región, Sakhalin Island (KP980319, KP980509, KP979954, KP980125); ***C. glareosa:*** Finland, Central Ostrobothnia, Kälviä (KP980343, KP980535, KP979976, KP980145); ***C. heleonastes:*** Canada, Yukon Territory, Halfway Lakes (KP980270, KP980458, KP979908, KP980085); ***C. kreczetoviczii:*** Russian Federation, Kamchatka Krai, Yelizovsky district (KP980354, KP980546, KP979987, KP980154); ***C. lachenalii:*** Spain, Lérida, Valle de Arán (KP980346, KP980538, KP979979, KP980148); ***C. loliacea:*** Finland, Oulun Pohjanmaa, Kiiminki (KP980248, KP980435, KP979888, KP980065); ***C. mackenziei:*** Russian Federation, Leningrad district, Lavansaari (KP980174, KP980357, KP979811, KP994952); ***C. marina:*** Canada, Northwest Territories, Mackenzie District (KP980345, KP980537, KP979978, KP980147); ***C. nemurensis:*** Russian Federation, Sakhalin Oblast, Korsakov district (KP980184, KP980368, KP979822, KP979998); ***C. tenuiflora:*** Canada, Yukon Territory, Aishihik Road (KP980321, KP980511, KP979956, KP980127); ***C. trisperma:*** USA, Pennsylvania, Jefferson County (KP980292, KP980482, KP979929, KP980105); ***C. ursina:*** Canada, Yukon Territory, Ivvavik National Park (KP980246, KP980433, KP979886, KP980063).

Outgroup species:

***C. arctiformis:*** Canada, British Columbia, Vancouver Island (KP980199, KP980384, KP979839, KP980012); ***C. bonanzensis:*** Canada, Yukon Territory, O’Brien Creek (KP980192, KP980377, KP979831, KP980006); ***C. canescens:*** Spain, Lérida, Valle de Arán (KP980309, KP980499, KP979944, KP980117); ***C. lapponica:*** Finland, Pohjois-Pohjanmaa, Haukipudas (KP980332, KP980523, KP979963, KP980133); ***C. praeceptorum:*** USA, California, Shasta County (KP980291, KP980481, KP979928, KP980104); ***C. pseudololiacea:*** Russian Federation, Sakhalin region, Chirpoi Island (KP980269, KP980457, KP979907, KP980084); ***C. traiziscana:*** Russian Federation, Sakhalin region, Sakhalin Island (KP980222, KP980409, KP979865, KP980039).
