# Supplementary material for: Cryptic Species Due to Hybridization: A Combined Approach to Describe a New Species (Carex: Cyperaceae)
Source: PLoS One. 2016 Dec 14;11(12):e0166949. doi: 10.1371/journal.pone.0166949 (PMC5156347; doi:10.1371/journal.pone.0166949)
Supplement: S2 File — Results derived from the analyses implemented in IBM SPSS Statistics v.20 (IBM Inc., Chicago, IL, USA) using morphological variables measured in Carex furva s.l. (A) Results of the analyses including all 21 measured variables and the hybrid population. (B) Results of the PCA analysis including nine selected variables and the hybrid population. (C) Results including all 21 variables and excluding the hybrid population. (D) Results of the analysis using 11 selected variables and excluding the hybrid population. (DOC) [file pone.0166949.s005.doc]

Supporting information

S2 File. Results from Kaiser-Meyer-Olkin (KMO), Bartlett's tests and principal component analyses (PCA).

(A) Results of the analyses including all 21 measured variables and the hybrid population in Sierra Nevada. Includes CLMW, INFL, INFW, USPIKA, SLSPIKA, LSPIKA, SPIKL, SPIKW, PERL, PERW, PERBKL, PERMWD, PERSTL, PSCLL, PSCLW, MINHYAL, MAXHYAL, ACHL, ACHW, SPKN and PERIGTHN.

KMO and Bartlett's Test	
Kaiser-Meyer-Olkin Measure of Sampling Adequacy.	0.692	
Bartlett's Test of Sphericity	Approx. Chi-Square	798.444	
	df	210	
	Sig.	0.000	

Communalities	
	Initial	Extraction	
CLMW	1.000	0.682	
INFL	1.000	0.836	
INFW	1.000	0.816	
USPIKA	1.000	0.643	
SLSPIKA	1.000	0.687	
LSPIKA	1.000	0.755	
SPIKL	1.000	0.641	
SPIKW	1.000	0.662	
PERL	1.000	0.842	
PERW	1.000	0.738	
PERBKL	1.000	0.787	
PERMWD	1.000	0.657	
PERSTL	1.000	0.589	
PSCLL	1.000	0.621	
PSCLW	1.000	0.674	
MINHYAL	1.000	0.681	
MAXHYAL	1.000	0.653	
ACHL	1.000	0.676	
ACHW	1.000	0.724	
SPKN	1.000	0.713	
PERIGTHN	1.000	0.378	
Extraction Method: Principal Component Analysis.	


Total Variance Explained	
Component	Initial Eigenvalues	Extraction Sums of Squared Loadings	
	Total	% of Variance	Cumulative %	Total	% of Variance	Cumulative %	
1	4.418	21.038	21.038	4.418	21.038	21.038	
2	3.310	15.762	36.800	3.310	15.762	36.800	
3	1.783	8.492	45.292	1.783	8.492	45.292	
4	1.481	7.054	52.346	1.481	7.054	52.346	
5	1.246	5.932	58.278	1.246	5.932	58.278	
6	1.130	5.383	63.662	1.130	5.383	63.662	
7	1.087	5.175	68.837	1.087	5.175	68.837	
8	0.983	4.683	73.520				
9	0.806	3.840	77.360				
10	0.730	3.475	80.834				
11	0.668	3.181	84.016				
12	0.525	2.501	86.517				
13	0.519	2.469	88.986				
14	0.460	2.191	91.177				
15	0.414	1.972	93.149				
16	0.345	1.644	94.793				
17	0.307	1.462	96.255				
18	0.260	1.237	97.492				
19	0.225	1.070	98.562				
20	0.188	0.895	99.458				
21	0.114	0.542	100.000				
Extraction Method: Principal Component Analysis.	
	

Component Matrixa	
	Component	
	1	2	3	4	5	6	7	
CLMW	0.674	0.353	0.200	-0.136	0.211	0.000	0.002	
INFL	-0.041	0.790	0.096	-0.020	0.176	-0.148	0.385	
INFW	0.637	0.396	-0.084	-0.465	0.089	-0.109	-0.100	
USPIKA	-0.184	-0.073	0.019	-0.247	0.353	0.645	0.045	
SLSPIKA	-0.353	0.152	0.042	0.452	0.472	0.129	-0.306	
LSPIKA	-0.413	0.102	0.232	0.492	0.000	0.052	0.525	
SPIKL	0.294	0.639	-0.093	0.028	0.323	0.165	0.076	
SPIKW	0.652	0.159	-0.233	-0.033	0.192	-0.221	-0.266	
PERL	0.832	-0.327	-0.036	0.165	0.060	-0.028	0.098	
PERW	0.320	0.323	0.559	0.067	-0.421	0.121	-0.152	
PERBKL	0.790	-0.244	-0.139	-0.008	-0.032	0.116	0.263	
PERMWD	0.372	0.055	0.347	0.480	0.401	-0.030	-0.053	
PERSTL	0.579	-0.239	-0.061	0.157	-0.215	0.287	0.200	
PSCLL	0.503	0.239	-0.400	0.333	-0.110	0.168	0.011	
PSCLW	0.282	0.596	-0.204	0.006	-0.258	0.346	0.110	
MINHYAL	-0.152	0.476	-0.445	0.338	-0.152	-0.237	-0.200	
MAXHYAL	-0.263	0.514	-0.455	0.228	-0.247	-0.002	-0.013	
ACHL	0.577	-0.371	0.136	0.309	0.036	-0.298	-0.032	
ACHW	0.207	0.242	0.618	0.160	-0.347	0.134	-0.276	
SPKN	-0.097	0.572	0.357	-0.225	0.024	-0.347	0.278	
PERIGTHN	-0.116	0.417	0.131	-0.041	0.017	0.205	-0.359	
Extraction Method: Principal Component Analysis.	
a. 7 components extracted.	


Principal Component Analysis (PCA) scatter plot of the first two principal components using all morphological variables and including the hybrid population in Sierra Nevada. Circles represent Carex furva s.s., triangles for C. lucennoiberica, and squares for specimens of the hybrid population. Colors indicate the mountain range where the specimens were collected, where C1 = Serra da Estrela; C2 = Sierra de Béjar; C3 = Sierra del Barco; C4 = Picos de Gredos; C5 = Sierra de Guadarrama; N1 = Sierra Segundera; N2 = Sierra del Cornón; N3 = Fuentes Carrionas (Curavacas); S1 (C. furva s.s.) and S2 (hybrids) = Sierra Nevada).

(B) Results of the PCA analysis including 9 selected variables and the hybrid population in Sierra Nevada. Principal component analysis includes those 9 variables with more than or at least 0.6 weight in components 1 to 7 in component matrix obtained using all 21 variables (see above): CLMW, INFL, INFW, USPIKA, SPIKL, SPIKW, PERL, PERBKL and ACHW.

KMO and Bartlett's Test	
Kaiser-Meyer-Olkin Measure of Sampling Adequacy.	0.731	
Bartlett's Test of Sphericity	Approx. Chi-Square	279.695	
	df	36	
	Sig.	0.000	

Communalities	
	Initial	Extraction	
CLMW	1.000	0.708	
INFW	1.000	0.614	
SPIKW	1.000	0.574	
PERL	1.000	0.831	
PERBKL	1.000	0.788	
INFL	1.000	0.760	
SPIKL	1.000	0.705	
ACHW	1.000	0.955	
USPIKA	1.000	0.969	
Extraction Method: Principal Component Analysis.	

Total Variance Explained	
Component	Initial Eigenvalues	Extraction Sums of Squared Loadings	
	Total	% of Variance	Cumulative %	Total	% of Variance	Cumulative %	
1	3.092	34.352	34.352	3.092	34.352	34.352	
2	1.788	19.863	54.215	1.788	19.863	54.215	
3	1.020	11.334	65.549	1.020	11.334	65.549	
4	1.005	11.168	76.717	1.005	11.168	76.717	
5	0.620	6.886	83.603				
6	0.476	5.292	88.895				
7	0.439	4.876	93.771				
8	0.361	4.008	97.779				
9	0.200	2.221	100.000				
Extraction Method: Principal Component Analysis.	


Component Matrixa	
	Component	
	1	2	3	4	
CLMW	0.797	0.193	0.188	-0.008	
INFW	0.757	0.190	-0.004	0.068	
SPIKW	0.739	-0.065	-0.088	-0.127	
PERL	0.674	-0.611	-0.002	0.060	
PERBKL	0.695	-0.523	-0.048	0.172	
INFL	0.222	0.811	-0.229	0.006	
SPIKL	0.540	0.594	-0.159	0.187	
ACHW	0.170	0.216	0.884	-0.313	
USPIKA	-0.177	0.065	0.339	0.905	
Extraction Method: Principal Component Analysis.	
a. 4 components extracted.	


Principal Component Analysis (PCA) scatter plot of the first two principal components using selected variables and including the hybrid population in Sierra Nevada. Circles represent Carex furva s.s., triangles for C. lucennoiberica, and squares for specimens of the hybrid population. Colors indicate the mountain range where the specimens were collected, where C1 = Serra da Estrela; C2 = Sierra de Béjar; C3 = Sierra del Barco; C4 = Picos de Gredos; C5 = Sierra de Guadarrama; N1 = Sierra Segundera; N2 = Sierra del Cornón; N3 = Fuentes Carrionas (Curavacas); S1 (C. furva s.s.) and S2 (hybrid) = Sierra Nevada.

(C) Results of the analyses including all 21 measured variables and excluding the hybrid population in Sierra Nevada. Includes CLMW, INFL, INFW, USPIKA, SLSPIKA, LSPIKA, SPIKL, SPIKW, PERL, PERW, PERBKL, PERMWD, PERSTL, PSCLL, PSCLW, MINHYAL, MAXHYAL, ACHL, ACHW, SPKN and PERIGTHN.

KMO and Bartlett's Test	
Kaiser-Meyer-Olkin Measure of Sampling Adequacy.	0.697	
Bartlett's Test of Sphericity	Approx. Chi-Square	719.522	
	df	210	
	Sig.	0.000	

Communalities	
	Initial	Extraction	
CLMW	1.000	0.682	
INFL	1.000	0.765	
INFW	1.000	0.798	
USPIKA	1.000	0.664	
SLSPIKA	1.000	0.669	
LSPIKA	1.000	0.546	
SPIKL	1.000	0.640	
SPIKW	1.000	0.647	
PERL	1.000	0.822	
PERW	1.000	0.728	
PERBKL	1.000	0.735	
PERMWD	1.000	0.641	
PERSTL	1.000	0.555	
PSCLL	1.000	0.716	
PSCLW	1.000	0.676	
MINHYAL	1.000	0.653	
MAXHYAL	1.000	0.640	
ACHL	1.000	0.688	
ACHW	1.000	0.685	
SPKN	1.000	0.807	
PERIGTHN	1.000	0.761	
Extraction Method: Principal Component Analysis.	


Total Variance Explained	
Component	Initial Eigenvalues	Extraction Sums of Squared Loadings	
	Total	% of Variance	Cumulative %	Total	% of Variance	Cumulative %	
1	4.583	21.822	21.822	4.583	21.822	21.822	
2	3.291	15.673	37.495	3.291	15.673	37.495	
3	1.802	8.582	46.077	1.802	8.582	46.077	
4	1.389	6.615	52.692	1.389	6.615	52.692	
5	1.265	6.024	58.716	1.265	6.024	58.716	
6	1.120	5.333	64.049	1.120	5.333	64.049	
7	1.066	5.077	69.126	1.066	5.077	69.126	
8	0.976	4.646	73.773				
9	0.809	3.850	77.623				
10	0.735	3.499	81.122				
11	0.655	3.121	84.243				
12	0.562	2.678	86.921				
13	0.489	2.331	89.251				
14	0.457	2.178	91.429				
15	0.407	1.940	93.369				
16	0.333	1.584	94.953				
17	0.286	1.360	96.313				
18	0.246	1.174	97.487				
19	0.228	1.084	98.571				
20	0.184	0.877	99.448				
21	0.116	0.552	100.000				
Extraction Method: Principal Component Analysis.	

Component Matrixa	
	Component	
	1	2	3	4	5	6	7	
CLMW	0.679	0.323	0.229	-0.177	0.156	-0.010	-0.084	
INFL	-0.089	0.801	0.101	-0.166	0.071	-0.097	0.252	
INFW	0.676	0.373	-0.121	-0.401	0.001	-0.149	-0.067	
USPIKA	-0.162	-0.048	0.136	-0.444	0.144	0.598	0.203	
SLSPIKA	-0.390	0.149	0.169	0.241	0.628	0.112	-0.036	
LSPIKA	-0.483	0.108	0.260	0.387	0.026	0.214	0.193	
SPIKL	0.249	0.649	-0.016	-0.060	0.282	0.203	-0.179	
SPIKW	0.694	0.138	-0.190	0.022	0.198	-0.241	-0.113	
PERL	0.858	-0.263	-0.032	0.089	0.086	0.008	0.023	
PERW	0.283	0.320	0.547	0.144	-0.445	-0.027	-0.162	
PERBKL	0.799	-0.212	-0.128	-0.034	-0.085	0.158	0.049	
PERMWD	0.363	0.146	0.428	0.240	0.495	0.018	0.042	
PERSTL	0.587	-0.167	-0.021	0.256	-0.152	0.291	0.094	
PSCLL	0.493	0.319	-0.371	0.292	0.005	0.296	0.247	
PSCLW	0.236	0.611	-0.191	0.062	-0.267	0.367	0.027	
MINHYAL	-0.157	0.497	-0.398	0.376	0.050	-0.257	0.113	
MAXHYAL	-0.263	0.525	-0.417	0.308	-0.151	-0.054	0.026	
ACHL	0.563	-0.354	0.182	0.300	0.136	-0.272	0.172	
ACHW	0.170	0.167	0.667	0.272	-0.318	0.013	0.086	
SPKN	-0.104	0.585	0.277	-0.335	-0.057	-0.302	0.412	
PERIGTHN	-0.142	0.413	0.110	0.078	-0.017	0.079	-0.738	
Extraction Method: Principal Component Analysis.	
a. 7 components extracted.	


Principal Component Analysis (PCA) scatter plot of the first two principal components using all morphological variables and excluding the hybrid population in Sierra Nevada. Circles represent Carex furva s.s. and triangles for C. lucennoiberica. Colors indicate the mountain range where the specimens were collected, where C1 = Serra da Estrela; C2 = Sierra de Béjar; C3 = Sierra del Barco; C4 = Picos de Gredos; C5 = Sierra de Guadarrama; N1 = Sierra Segundera; N2 = Sierra del Cornón; N3 = Fuentes Carrionas (Curavacas); S1 = Sierra Nevada.

(D) Results of the PCA analysis including 11 selected variables and excluding the hybrid population in Sierra Nevada. Principal component analysis includes those 11 variables with more than or at least 0.6 weight in components 1 to 7 in component matrix obtained using all 21 variables (see above): CLMW, INFL, INFW, SLSPIKA, SPIKL, SPIKW, PERL, PERBKL, PSCLW, ACHW and PERIGTHN.

KMO and Bartlett's Test	
Kaiser-Meyer-Olkin Measure of Sampling Adequacy.	0.757	
Bartlett's Test of Sphericity	Approx. Chi-Square	311.356	
	df	55	
	Sig.	0.000	

Communalities	
	Initial	Extraction	
CLMW	1.000	0.651	
INFW	1.000	0.658	
SPIKW	1.000	0.534	
PERL	1.000	0.789	
PERBKL	1.000	0.753	
INFL	1.000	0.650	
SPIKL	1.000	0.655	
PSCLW	1.000	0.420	
ACHW	1.000	0.938	
SLSPIKA	1.000	0.306	
PERIGTHN	1.000	0.283	
Extraction Method: Principal Component Analysis.	
Total Variance Explained	
Component	Initial Eigenvalues	Extraction Sums of Squared Loadings	
	Total	% of Variance	Cumulative %	Total	% of Variance	Cumulative %	
1	3.374	30.676	30.676	3.374	30.676	30.676	
2	2.223	20.212	50.889	2.223	20.212	50.889	
3	1.039	9.447	60.336	1.039	9.447	60.336	
4	0.981	8.918	69.253				
5	0.857	7.795	77.048				
6	0.606	5.505	82.553				
7	0.575	5.229	87.782				
8	0.436	3.963	91.745				
9	0.395	3.595	95.340				
10	0.325	2.952	98.292				
11	0.188	1.708	100.000				
Extraction Method: Principal Component Analysis.	
Component Matrixa	
	Component	
	1	2	3	
CLMW	0.762	0.199	0.176	
INFW	0.792	0.157	-0.077	
SPIKW	0.728	-0.054	-0.031	
PERL	0.741	-0.490	-0.010	
PERBKL	0.737	-0.449	-0.091	
INFL	0.145	0.770	-0.190	
SPIKL	0.455	0.644	-0.182	
PSCLW	0.366	0.534	0.029	
ACHW	0.115	0.135	0.952	
SLSPIKA	-0.411	0.368	-0.038	
PERIGTHN	-0.014	0.518	0.121	
Extraction Method: Principal Component Analysis.	
a. 3 components extracted.	


Principal Component Analysis (PCA) scatter plot of the first two principal components using selected variables and excluding the hybrid population in Sierra Nevada. Circles represent Carex furva s.s. and triangles for C. lucennoiberica. Colors indicate the mountain range where the specimens were collected, where C1 = Serra da Estrela; C2 = Sierra de Béjar; C3 = Sierra del Barco; C4 = Picos de Gredos; C5 = Sierra de Guadarrama; N1 = Sierra Segundera; N2 = Sierra del Cornón; N3 = Fuentes Carrionas (Curavacas); S1 = Sierra Nevada.
