# Supplementary material for: Cryptic Species Due to Hybridization: A Combined Approach to Describe a New Species (Carex: Cyperaceae)
Source: PLoS One. 2016 Dec 14;11(12):e0166949. doi: 10.1371/journal.pone.0166949 (PMC5156347; doi:10.1371/journal.pone.0166949)
Supplement: S3 File — Results derived from the analyses implemented in IBM SPSS Statistics v.20 (IBM Inc., Chicago, IL, USA) using morphological variables measured in Carex furva s.l. Variables included: CLMW, INFL, INFW, USPIKA, SLSPIKA, LSPIKA, SPIKL, SPIKW, PERL, PERW, PERBKL, PERMWD, PERSTL, PSCLL, PSCLW, MINHYAL, MAXHYAL, ACHL, ACHW, SPKN and PERIGTHN. (DOCX) [file pone.0166949.s006.docx]

**Supporting Information**

**S3 File. Discriminant function analysis (DFA) results.**

**Summary of Canonical Discriminant Functions**

| **Eigenvalues** | | | | |
| --- | --- | --- | --- | --- |
| Function | Eigenvalue | % of Variance | Cumulative % | Canonical Correlation |
| 1 | 4.962^a^ | 100.0 | 100.0 | 0.912 |
| a. First 1 canonical discriminant functions were used in the analysis. | | | | |

| **Wilks' Lambda** | | | | |
| --- | --- | --- | --- | --- |
| Test of Function(s) | Wilks' Lambda | Chi-square | df | Sig. |
| 1 | 0.168 | 91.952 | 21 | 0.000 |

| **Standardized Canonical Discriminant Function Coefficients** | |
| --- | --- |
|  | Function |
|  | 1 |
| CLMW | 0.199 |
| INFL | -0.610 |
| INFW | 0.454 |
| USPIKA | 0.114 |
| SLSPIKA | -0.252 |
| LSPIKA | -0.005 |
| SPIKL | -0.225 |
| SPIKW | 0.357 |
| PERL | 0.704 |
| PERW | 0.007 |
| PERBKL | -0.159 |
| PERMWD | -0.367 |
| PERSTL | 0.233 |
| PSCLL | 0.255 |
| PSCLW | -0.217 |
| MINHYAL | -0.169 |
| MAXHYAL | 0.219 |
| ACHL | 0.222 |
| ACHW | -0.215 |
| SPKN | -0.084 |
| PERIGTHN | -0.054 |

| **Functions at Group Centroids** | |
| --- | --- |
| species | Function |
|  | 1 |
| *C. lucennoiberica* | -1.266 |
| *C. furva s.s.* | 3.798 |
| Unstandardized canonical discriminant functions evaluated at group means | |

| **Structure Matrix** | |
| --- | --- |
|  | Function |
|  | 1 |
| PERL | 0.577 |
| PERBKL | 0.403 |
| ACHL | 0.295 |
| SPIKW | 0.290 |
| SLSPIKA | -0.241 |
| INFL | -0.238 |
| INFW | 0.231 |
| CLMW | 0.224 |
| PERSTL | 0.219 |
| LSPIKA | -0.212 |
| PSCLL | 0.205 |
| SPKN | -0.175 |
| PERIGTHN | -0.117 |
| MAXHYAL | -0.086 |
| MINHYAL | -0.063 |
| SPIKL | -0.040 |
| ACHW | -0.033 |
| USPIKA | 0.027 |
| PERW | -0.025 |
| PERMWD | 0.010 |
| PSCLW | -0.007 |
| Pooled within-groups correlations between discriminating variables and standardized canonical discriminant functions  Variables ordered by absolute size of correlation within function. | |

| **Classification Results^a,b,d^** | | | | | | |
| --- | --- | --- | --- | --- | --- | --- |
|  |  |  | species | Predicted Group Membership | | Total |
|  |  |  |  | *C. lucennoiberica* | *C. furva s.s.* |  |
| Cases Selected | Original | Count | *C. lucennoiberica* | 48 | 0 | 48 |
|  |  |  | *C. furva s.s.* | 0 | 16 | 16 |
|  |  | % | *C. lucennoiberica* | 100.0 | 0.0 | 100.0 |
|  |  |  | *C. furva s.s.* | 0.0 | 100.0 | 100.0 |
|  | Cross-validated^c^ | Count | *C. lucennoiberica* | 46 | 2 | 48 |
|  |  |  | *C. furva s.s.* | 3 | 13 | 16 |
|  |  | % | *C. lucennoiberica* | 95.8 | 4.2 | 100.0 |
|  |  |  | *C. furva s.s.* | 18.8 | 81.3 | 100.0 |
| Cases Not Selected | Original | Count | *C. lucennoiberica* | 18 | 2 | 20 |
|  |  |  | *C. furva s.s.* | 0 | 5 | 5 |
|  |  |  | Hybrid population | 8 | 3 | 11 |
|  |  | % | *C. lucennoiberica* | 90.0 | 10.0 | 100.0 |
|  |  |  | *C. furva s.s.* | 0.0 | 100.0 | 100.0 |
|  |  |  | Hybrid population | 72.7 | 27.3 | 100.0 |
| a. 100.0% of selected original grouped cases correctly classified. | | | | | | |
| b. 92.0% of unselected original grouped cases correctly classified. | | | | | | |
| c. Cross validation is done only for those cases in the analysis. In cross validation, each case is classified by the functions derived from all cases other than that case. | | | | | | |
| d. 92.2% of selected cross-validated grouped cases correctly classified. | | | | | | |
