# Supplementary material for: Cryptic Species Due to Hybridization: A Combined Approach to Describe a New Species (Carex: Cyperaceae)
Source: PLoS One. 2016 Dec 14;11(12):e0166949. doi: 10.1371/journal.pone.0166949 (PMC5156347; doi:10.1371/journal.pone.0166949)
Supplement: S4 File — Results derived from the analyses implemented in IBM SPSS Statistics v.20 (IBM Inc., Chicago, IL, USA) using 21 morphological variables: CLMW, INFL, INFW, USPIKA, SLSPIKA, LSPIKA, SPIKL, SPIKW, PERL, PERW, PERBKL, PERMWD, PERSTL, PSCLL, PSCLW, MINHYAL, MAXHYAL, ACHL, ACHW, SPKN and PERIGTHN. (DOCX) [file pone.0166949.s007.docx]

**Supporting Information**

**S4 File. Test of normality, ANOVA and Mann-Whitney U test results**.

| **Tests of Normality** | | | | | | |
| --- | --- | --- | --- | --- | --- | --- |
|  | Kolmogorov-Smirnov^a^ | | | Shapiro-Wilk | | |
|  | Statistic | df | Sig. | Statistic | df | Sig. |
| CLMW | 0.128 | 100 | 0.000 | 0.937 | 100 | 0.000 |
| INFL | 0.101 | 100 | 0.014 | 0.980 | 100 | 0.145 |
| INFW | 0.086 | 100 | 0.064 | 0.979 | 100 | 0.118 |
| USPIKA | 0.202 | 100 | 0.000 | 0.793 | 100 | 0.000 |
| SLSPIKA | 0.113 | 100 | 0.003 | 0.946 | 100 | 0.000 |
| LSPIKA | 0.077 | 100 | 0.150 | 0.980 | 100 | 0.133 |
| SPIKL | 0.100 | 100 | 0.015 | 0.979 | 100 | 0.108 |
| SPIKW | 0.061 | 100 | 0.200^*^ | 0.985 | 100 | 0.303 |
| PERL | 0.115 | 100 | 0.002 | 0.950 | 100 | 0.001 |
| PERW | 0.091 | 100 | 0.039 | 0.978 | 100 | 0.098 |
| PERBKL | 0.100 | 100 | 0.015 | 0.983 | 100 | 0.235 |
| PERMWD | 0.068 | 100 | 0.200^*^ | 0.979 | 100 | 0.118 |
| PERSTL | 0.095 | 100 | 0.027 | 0.972 | 100 | 0.031 |
| PSCLL | 0.053 | 100 | 0.200^*^ | 0.982 | 100 | 0.181 |
| PSCLW | 0.065 | 100 | 0.200^*^ | 0.988 | 100 | 0.524 |
| MINHYAL | 0.404 | 100 | 0.000 | 0.465 | 100 | 0.000 |
| MAXHYAL | 0.268 | 100 | 0.000 | 0.798 | 100 | 0.000 |
| ACHL | 0.063 | 100 | 0.200^*^ | 0.991 | 100 | 0.708 |
| ACHW | 0.121 | 100 | 0.001 | 0.955 | 100 | 0.002 |
| SPKN | 0.216 | 100 | 0.000 | 0.902 | 100 | 0.000 |
| PERIGTHN | 0.507 | 100 | 0.000 | 0.439 | 100 | 0.000 |
| *. This is a lower bound of the true significance. | | | | | | |
| a. Lilliefors Significance Correction | | | | | | |

| **Test of Homogeneity of Variances** | | | | |
| --- | --- | --- | --- | --- |
|  | Levene Statistic | df1 | df2 | Sig. |
| CLMW | 2.821 | 1 | 87 | 0.097 |
| INFL | 5.267 | 1 | 87 | 0.024 |
| INFW | 2.413 | 1 | 87 | 0.124 |
| USPIKA | 1.098 | 1 | 87 | 0.298 |
| SLSPIKA | 5.373 | 1 | 87 | 0.023 |
| LSPIKA | 5.688 | 1 | 87 | 0.019 |
| SPIKL | 0.425 | 1 | 87 | 0.516 |
| SPIKW | 3.981 | 1 | 87 | 0.049 |
| PERL | 0.858 | 1 | 87 | 0.357 |
| PERW | 0.143 | 1 | 87 | 0.706 |
| PERBKL | 0.233 | 1 | 87 | 0.630 |
| PERMWD | 1.543 | 1 | 87 | 0.218 |
| PERSTL | 1.389 | 1 | 87 | 0.242 |
| PSCLL | 0.123 | 1 | 87 | 0.727 |
| PSCLW | 10.021 | 1 | 87 | 0.002 |
| MINHYAL | 6.893 | 1 | 87 | 0.010 |
| MAXHYAL | 8.201 | 1 | 87 | 0.005 |
| ACHL | 0.443 | 1 | 87 | 0.507 |
| ACHW | 2.322 | 1 | 87 | 0.131 |
| SPKN | 3.492 | 1 | 87 | 0.065 |
| PERIGTHN | 27.772 | 1 | 87 | 0.000 |

| **ANOVA** | | | | | | |
| --- | --- | --- | --- | --- | --- | --- |
|  | | Sum of Squares | df | Mean Square | F | Sig. |
| CLMW | Between Groups | 0.190 | 1 | 0.190 | 5.144 | 0.026 |
|  | Within Groups | 3.208 | 87 | 0.037 |  |  |
|  | Total | 3.398 | 88 |  |  |  |
| INFL | Between Groups | 76.550 | 1 | 76.550 | 31.493 | 0.000 |
|  | Within Groups | 211.468 | 87 | 2.431 |  |  |
|  | Total | 288.017 | 88 |  |  |  |
| INFW | Between Groups | 19.024 | 1 | 19.024 | 12.255 | 0.001 |
|  | Within Groups | 135.057 | 87 | 1.552 |  |  |
|  | Total | 154.081 | 88 |  |  |  |
| USPIKA | Between Groups | 50.603 | 1 | 50.603 | 0.411 | 0.523 |
|  | Within Groups | 10700.835 | 87 | 122.998 |  |  |
|  | Total | 10751.438 | 88 |  |  |  |
| SLSPIKA | Between Groups | 6469.316 | 1 | 6469.316 | 28.894 | 0.000 |
|  | Within Groups | 19479.403 | 87 | 223.901 |  |  |
|  | Total | 25948.719 | 88 |  |  |  |
| LSPIKA | Between Groups | 3315.053 | 1 | 3315.053 | 17.149 | 0.000 |
|  | Within Groups | 16817.756 | 87 | 193.308 |  |  |
|  | Total | 20132.809 | 88 |  |  |  |
| SPIKL | Between Groups | 1.932 | 1 | 1.932 | 3.429 | 0.067 |
|  | Within Groups | 49.019 | 87 | 0.563 |  |  |
|  | Total | 50.951 | 88 |  |  |  |
| SPIKW | Between Groups | 7.937 | 1 | 7.937 | 25.713 | 0.000 |
|  | Within Groups | 26.853 | 87 | 0.309 |  |  |
|  | Total | 34.790 | 88 |  |  |  |
| PERL | Between Groups | 3.868 | 1 | 3.868 | 98.973 | 0.000 |
|  | Within Groups | 3.400 | 87 | 0.039 |  |  |
|  | Total | 7.268 | 88 |  |  |  |
| PERW | Between Groups | 0.004 | 1 | 0.004 | 0.239 | 0.626 |
|  | Within Groups | 1.342 | 87 | 0.015 |  |  |
|  | Total | 1.345 | 88 |  |  |  |
| PERBKL | Between Groups | 0.545 | 1 | 0.545 | 51.939 | 0.000 |
|  | Within Groups | 0.912 | 87 | 0.010 |  |  |
|  | Total | 1.457 | 88 |  |  |  |
| PERMWD | Between Groups | 0.003 | 1 | 0.003 | 0.200 | 0.656 |
|  | Within Groups | 1.232 | 87 | 0.014 |  |  |
|  | Total | 1.235 | 88 |  |  |  |
| PERSTL | Between Groups | 0.406 | 1 | 0.406 | 22.271 | 0.000 |
|  | Within Groups | 1.585 | 87 | 0.018 |  |  |
|  | Total | 1.991 | 88 |  |  |  |
| PSCLL | Between Groups | 0.319 | 1 | 0.319 | 5.950 | 0.017 |
|  | Within Groups | 4.660 | 87 | 0.054 |  |  |
|  | Total | 4.979 | 88 |  |  |  |
| PSCLW | Between Groups | 0.013 | 1 | 0.013 | 0.326 | 0.570 |
|  | Within Groups | 3.436 | 87 | 0.039 |  |  |
|  | Total | 3.449 | 88 |  |  |  |
| MINHYAL | Between Groups | 0.011 | 1 | 0.011 | 2.261 | 0.136 |
|  | Within Groups | 0.417 | 87 | 0.005 |  |  |
|  | Total | 0.428 | 88 |  |  |  |
| MAXHYAL | Between Groups | 0.984 | 1 | 0.984 | 5.106 | 0.026 |
|  | Within Groups | 16.760 | 87 | 0.193 |  |  |
|  | Total | 17.744 | 88 |  |  |  |
| ACHL | Between Groups | 0.298 | 1 | 0.298 | 26.676 | 0.000 |
|  | Within Groups | 0.973 | 87 | 0.011 |  |  |
|  | Total | 1.272 | 88 |  |  |  |
| ACHW | Between Groups | 0.007 | 1 | 0.007 | 0.807 | 0.372 |
|  | Within Groups | 0.743 | 87 | 0.009 |  |  |
|  | Total | 0.750 | 88 |  |  |  |
| SPKN | Between Groups | 10.789 | 1 | 10.789 | 12.228 | 0.001 |
|  | Within Groups | 76.761 | 87 | 0.882 |  |  |
|  | Total | 87.551 | 88 |  |  |  |
| PERIGTHN | Between Groups | 0.680 | 1 | 0.680 | 4.511 | 0.037 |
|  | Within Groups | 13.118 | 87 | 0.151 |  |  |
|  | Total | 13.798 | 88 |  |  |  |

**Mann-Whitney U Test**

| **Ranks** | | | | |
| --- | --- | --- | --- | --- |
|  | species | N | Mean Rank | Sum of Ranks |
| CLMW | *C. lucennoiberica* | 68 | 42.32 | 2877.50 |
|  | *C. furva s.s.* | 21 | 53.69 | 1127.50 |
|  | Total | 89 |  |  |
| INFL | *C. lucennoiberica* | 68 | 52.53 | 3572.00 |
|  | *C. furva s.s.* | 21 | 20.62 | 433.00 |
|  | Total | 89 |  |  |
| INFW | *C. lucennoiberica* | 68 | 39.79 | 2705.50 |
|  | *C. furva s.s.* | 21 | 61.88 | 1299.50 |
|  | Total | 89 |  |  |
| USPIKA | *C. lucennoiberica* | 68 | 45.51 | 3095.00 |
|  | *C. furva s.s.* | 21 | 43.33 | 910.00 |
|  | Total | 89 |  |  |
| SLSPIKA | *C. lucennoiberica* | 68 | 51.61 | 3509.50 |
|  | *C. furva s.s.* | 21 | 23.60 | 495.50 |
|  | Total | 89 |  |  |
| LSPIKA | *C. lucennoiberica* | 68 | 50.91 | 3462.00 |
|  | *C. furva s.s.* | 21 | 25.86 | 543.00 |
|  | Total | 89 |  |  |
| SPIKL | *C. lucennoiberica* | 68 | 47.59 | 3236.00 |
|  | *C. furva s.s.* | 21 | 36.62 | 769.00 |
|  | Total | 89 |  |  |
| SPIKW | *C. lucennoiberica* | 68 | 38.71 | 2632.50 |
|  | *C. furva s.s.* | 21 | 65.36 | 1372.50 |
|  | Total | 89 |  |  |
| PERL | *C. lucennoiberica* | 68 | 35.40 | 2407.50 |
|  | *C. furva s.s.* | 21 | 76.07 | 1597.50 |
|  | Total | 89 |  |  |
| PERW | *C. lucennoiberica* | 68 | 45.36 | 3084.50 |
|  | *C. furva s.s.* | 21 | 43.83 | 920.50 |
|  | Total | 89 |  |  |
| PERBKL | *C. lucennoiberica* | 68 | 36.52 | 2483.50 |
|  | *C. furva s.s.* | 21 | 72.45 | 1521.50 |
|  | Total | 89 |  |  |
| PERMWD | *C. lucennoiberica* | 68 | 46.38 | 3153.50 |
|  | *C. furva s.s.* | 21 | 40.55 | 851.50 |
|  | Total | 89 |  |  |
| PERSTL | *C. lucennoiberica* | 68 | 38.96 | 2649.00 |
|  | *C. furva s.s.* | 21 | 64.57 | 1356.00 |
|  | Total | 89 |  |  |
| PSCLL | *C. lucennoiberica* | 68 | 41.20 | 2801.50 |
|  | *C. furva s.s.* | 21 | 57.31 | 1203.50 |
|  | Total | 89 |  |  |
| PSCLW | *C. lucennoiberica* | 68 | 45.88 | 3119.50 |
|  | *C. furva s.s.* | 21 | 42.17 | 885.50 |
|  | Total | 89 |  |  |
| MINHYAL | *C. lucennoiberica* | 68 | 47.29 | 3216.00 |
|  | *C. furva s.s.* | 21 | 37.57 | 789.00 |
|  | Total | 89 |  |  |
| MAXHYAL | *C. lucennoiberica* | 68 | 48.80 | 3318.50 |
|  | *C. furva s.s.* | 21 | 32.69 | 686.50 |
|  | Total | 89 |  |  |
| ACHL | *C. lucennoiberica* | 68 | 37.79 | 2569.50 |
|  | *C. furva s.s.* | 21 | 68.36 | 1435.50 |
|  | Total | 89 |  |  |
| ACHW | *C. lucennoiberica* | 68 | 46.82 | 3183.50 |
|  | *C. furva s.s.* | 21 | 39.12 | 821.50 |
|  | Total | 89 |  |  |
| SPKN | *C. lucennoiberica* | 68 | 49.71 | 3380.50 |
|  | *C. furva s.s.* | 21 | 29.74 | 624.50 |
|  | Total | 89 |  |  |
| PERIGTHN | *C. lucennoiberica* | 68 | 47.01 | 3196.50 |
|  | *C. furva s.s.* | 21 | 38.50 | 808.50 |
|  | Total | 89 |  |  |

**Test Statistics^a^**

|  | CLMW | INFL | INFW | USPIKA | SLSPIKA | LSPIKA | SPIKL | SPIKW | PERL | PERW | PERBKL |
| --- | --- | --- | --- | --- | --- | --- | --- | --- | --- | --- | --- |
| Mann-Whitney U | 531.500 | 202.000 | 359.500 | 679.000 | 264.500 | 312.000 | 538.000 | 286.500 | 61.500 | 689.500 | 137.500 |
| Wilcoxon W | 2877.500 | 433.000 | 2705.500 | 910.000 | 495.500 | 543.000 | 769.000 | 2632.500 | 2407.500 | 920.500 | 2483.500 |
| Z | -1.765 | -4.949 | -3.427 | -0.343 | -4.345 | -3.886 | -1.702 | -4.135 | -6.308 | -0.237 | -5.582 |
| Asymp. Sig. (2-tailed) | 0.078 | 0.000 | 0.001 | 0.732 | 0.000 | 0.000 | 0.089 | 0.000 | 0.000 | 0.812 | 0.000 |

|  | PERMWD | PERSTL | PSCLL | PSCLW | | MINHYAL | MAXHYAL | ACHL | ACHW | SPKN | PERIGTHN |
| --- | --- | --- | --- | --- | --- | --- | --- | --- | --- | --- | --- |
| Mann-Whitney U | 620.500 | 303.000 | 455.500 | 654.500 | | 558.000 | 455.500 | 223.500 | 590.500 | 393.500 | 577.500 |
| Wilcoxon W | 851.500 | 2649.000 | 2801.500 | 885.500 | | 789.000 | 686.500 | 2569.500 | 821.500 | 624.500 | 808.500 |
| Z | -0.905 | -3.977 | -2.499 | -0.575 | | -1.959 | -2.585 | -4.749 | -1.198 | -3.258 | -2.154 |
| Asymp. Sig. (2-tailed) | 0.365 | 0.000 | 0.012 | 0.565 | | 0.050 | 0.010 | 0.000 | 0.231 | 0.001 | 0.031 |
| a. Grouping Variable: species | | | | |  |  |  |  |  |  |  |
